# USP13 ameliorates nonalcoholic fatty liver disease through inhibiting the activation of TAK1

**DOI:** 10.1186/s12967-024-05465-4

**Published:** 2024-07-20

**Authors:** Min Tang, Han Cao, Yunqin Ma, Shuangshuang Yao, Xiaohui Wei, Yijiong Tan, Fang liu, Yongde Peng, Nengguang Fan

**Affiliations:** 1 Department of Endocrinology and Metabolism, Shanghai General Hospital, Shanghai Jiao Tong University School of Medicine, Shanghai, China; 2 Department of Endocrinology and Metabolism, Shanghai General Hospital of Nanjing Medical University, Shanghai, China; 3 Department of Endocrinology, Songjiang District Central Hospital, Shanghai, China; 4 Department of Clinical Pharmacy, Shanghai General Hospital, Shanghai Jiao Tong University School of Medicine, Shanghai, China

**Keywords:** Ubiquitin specific protease 13, Transforming growth factor beta-activated Kinase 1, Nonalcoholic fatty liver disease, Ubiquitination

## Abstract

**Background:**

The molecular mechanisms underlying nonalcoholic fatty liver disease (NAFLD) remain to be fully elucidated. Ubiquitin specific protease 13 (USP13) is a critical participant in inflammation-related signaling pathways, which are linked to NAFLD. Herein, the roles of USP13 in NAFLD and the underlying mechanisms were investigated.

**Methods:**

L02 cells and mouse primary hepatocytes were subjected to free fatty acid (FFA) to establish an in vitro model reflective of NAFLD. To prepare in vivo model of NAFLD, mice fed a high-fat diet (HFD) for 16 weeks and leptin-deficient (*ob/ob*) mice were used. USP13 overexpression and knockout (KO) strategies were employed to study the function of USP13 in NAFLD in mice.

**Results:**

The expression of USP13 was markedly decreased in both in vitro and in vivo models of NAFLD. USP13 overexpression evidently inhibited lipid accumulation and inflammation in FFA-treated L02 cells in vitro. Consistently, the in vivo experiments showed that USP13 overexpression ameliorated hepatic steatosis and metabolic disorders in HFD-fed mice, while its deficiency led to contrary outcomes. Additionally, inflammation was similarly attenuated by USP13 overexpression and aggravated by its deficiency in HFD-fed mice. Notably, overexpressing of USP13 also markedly alleviated hepatic steatosis and inflammation in *ob/ob* mice. Mechanistically, USP13 bound to transforming growth factor β-activated kinase 1 (TAK1) and inhibited K63 ubiquitination and phosphorylation of TAK1, thereby dampening downstream inflammatory pathways and promoting insulin signaling pathways. Inhibition of TAK1 activation reversed the exacerbation of NAFLD caused by USP13 deficiency in mice.

**Conclusions:**

Our findings indicate the protective role of USP13 in NAFLD progression through its interaction with TAK1 and inhibition the ubiquitination and phosphorylation of TAK1. Targeting the USP13-TAK1 axis emerges as a promising therapeutic strategy for NAFLD treatment.

**Supplementary Information:**

The online version contains supplementary material available at 10.1186/s12967-024-05465-4.

## Introduction

As a prevailing form of chronic liver disease, nonalcoholic fatty liver disease (NAFLD) affects approximately one-fourth of the global population [1]. The incidence of NAFLD has continued to increase in parallel with the prevalence of type 2 diabetes and obesity [2]. NAFLD includes simple liver steatosis, nonalcoholic steatohepatitis (NASH) and cirrhosis [3]. A previous study reported that 20%–27% of NAFLD patients have NASH, a progressive form of NAFLD that can develop into liver cirrhosis or even hepatocellular cancer over time [4]. As hepatic steatosis progresses, the inflammatory state is frequently followed by a disturbance in lipid metabolism [5]. The persistent inflammatory state could stimulate the development of insulin resistance, further prompting hepatic lipid accumulation [6]. The interplay among inflammation, insulin resistance, and hepatic lipid metabolic disorders is central to NAFLD progression [7]. However, the mechanism of NAFLD is yet not fully elucidated and rare drugs have been approved to cure NAFLD to date [8]. The pathologic progression of simple steatosis to NASH can be suppressed more efficiently with an understanding of the underlying pathogenic mechanism.

Ubiquitination is a type of post-translational modification critical to protein stability and proper functioning of signal transduction pathways that reversibly combines ubiquitin (Ub) with a target substrate [9]. De-ubiquitination is dependent on de-ubiquitinating enzyme (DUB) catalysis, which can clear Ub from target proteins. Ub-specific proteases (USPs) comprise a DUB subfamily with the largest scale. USP4 [10], USP10 [11], and USP18 [12] have been demonstrated to alleviate NAFLD by suppressing insulin resistance and inflammation-related signals [specifically, the nuclear factor kappa B (NF-κB) and mitogen-activated protein kinase (MAPK) signaling pathways]. In contrast, USP14 directly interacts with and stabilizes fatty acid synthase (FASN) to exacerbate liver steatosis, insulin resistance, and hyperglycemia [13]. USP20 stabilizes 3-hydroxy-3-methylglutaryl coenzyme A reductase (HMGCR) to increase liver cholesterol biosynthesis, which aggravates metabolic disorders [14]. USP13 is a USP family member that has been shown to have critical effects on regulating the cell cycle, repairing DNA damage, stimulating autophagy, differentiating myoblasts, and controlling the quality of the endoplasmic reticulum [15]. It was previously reported to attenuate osteoarthritis by inhibiting inflammation, oxidative stress, and apoptotic cell death [16]. Another study showed that USP13 stabilized the interleukin (IL)-1 related receptor to inhibit lung inflammation [17]. Moreover, USP13 has also been reported to interact with the cGAS-STING signaling pathway to regulate innate immunity [18]. Recently, USP13 has also found to be involved in liver inflammation [19]. However, the study involving USP13 function in metabolic disorders and NAFLD remains limited.

Because inflammation and insulin resistance are considered critical for NAFLD progression [20], signaling pathways, including insulin signaling cascades, the NF-κB signaling pathway, and the MAPK signaling pathway, have key roles in NAFLD [21, 22]. Transforming growth factor-β-activated kinase-1(TAK1), which belongs to the MAPK kinase kinase (MAP3K) family, is an essential activator of MAPK and NF-κB signaling pathways [23–25], and then promote the signaling cascades of pro-inflammatory cytokines and inhabit insulin signaling pathway, and these pathways contribute to liver inflammation, insulin resistance, and hepatic steatosis, all of which are hallmarks of NAFLD. Previous studies indicated TAK1 is a key element in regulating NAFLD in liver steatosis, insulin resistance, and inflammation [5, 8, 26], and its phosphorylation levels is influenced by the level of ubiquitination [27, 28]. USP4 and USP18, as members of the USP family, have been shown to interact directly with TAK1 and subsequently de-ubiquitinate TAK1 and inhabit its activation to alleviate NAFLD [10, 12].

In the present study, the role of USP13 in NAFLD and its underlying mechanisms was investigated. USP13 expression was reduced in the livers of NALFD mice. USP13 overexpression significantly improved insulin resistance, hepatic steatosis and inflammation in HFD-fed and *ob/ob* mice. Mechanistically, USP13 interacted with TAK1, repressing its K63-linked ubiquitination and phosphorylation, thereby modulating downstream inflammatory and insulin pathways. These findings suggest that targeting USP13 could offer a novel therapeutic approach for NAFLD, potentially improving clinical outcomes for patients with this condition.

## Material & methods

### Mice and diets

Specific pathogen-free (SPF) mice were used in in vivo assays. Leptin-deficient (*ob/ob*) and C57BL/6 male mice aged 6–8 weeks were purchased from GemPharmatech Co., Ltd. (Nanjing, China). HFD contains 45%–75% of total calories from fat is the routine model of obesity in rodents [29], which represents the natural development of NAFLD. Thus, the HFD (60 kcal% fat, 20% kcal protein and 20% kcal carbohydrate, D12492; Research Diets Inc., New Brunswick, NJ, USA) was administered to 8-week-old male mice to create a mouse model of NAFLD, while control mice were fed a normal maintenance diet (ND) for 16 weeks. To validate the NAFLD models, histological analysis and biochemical analyses were used. Specifically, mice with HFD-feeding for 10–12 weeks can develop steatosis, which is indicated by high lipid accumulation, hyperlipidemia, hypercholesterolemia, hyperinsulinemia, and glucose intolerance [30, 31]. However, when mice fed HFD for 16 weeks, the hepatocyte steatosis, Mallory–Denk bodies and ballooning occurs, and lower serum levels of the anti-inflammatory factors and elevated levels of fasting serum glucose are observed [32, 33]. Furthermore, *ob/ob* mice were fed a ND. Blood glucose levels and body weight were determined every 2 weeks during fasting.

To achieve hepatic USP13 overexpression in C57BL/6 and *ob/ob* mice, an adeno-associated virus 8 (AAV8) vector system with CMV promoter constructed by OBiO Technology Corp., Ltd. (Shanghai, China) was used to create AAV-USP13, which was delivered in vivo to mice via the tail vein to overexpress hepatic USP13. Controls were given AAV-green fluorescent protein (GFP) injection. Six-week-old USP13 knockout (USP13-KO) male mice and their corresponding wild-type (WT) littermates were supplied by Cyagen Biosciences, Inc. (Suzhou, China). The CRISPR-Cas9 technique and C57BL/6J background mice was used to prepare USP13-KO mice. 8-week HFD fed USP13-KO mice received an intraperitoneal injection of 5Z-7-Oxozeaenol (Cat# O9890-1 MG; 5 mg/kg; Sigma, St. Louis, MO) once a week for 8 weeks to suppress the activation of TAK1.

All mice were randomly assigned to the experimental and control groups, and raised in a standard SPF environment in the Animal Centre (Shanghai General Hospital, Shanghai, China) at a humidity of 55 ± 5% and a temperature of 22 ± 2 °C with a 12/12-h light/dark cycle. The mice bred in ventilated cages had access to water and food *adlibitum*. All animal care and experimental procedures were authorized by the Institutional Animal Care and Use Committee of Shanghai General Hospital (approval no. 2022AW010) and conducted in accordance with the Guide for the Care and Use of Laboratory Animals (https://www.ncbi.nlm.nih.gov/books/NBK54050/).

### Cell culture

In order to establish a baseline control for our in vitro experiments, primary hepatocytes used in the study were isolated from healthy and untreated C57BL/6 male mice aged 6–8 weeks by liver perfusion method. In short, after anesthesia, opened the abdominal cavity of mice, and used liver perfusion medium and liver digest medium to perfuse the liver though the portal vein. Then, 100 μm steel mesh was performed to filter the digested liver tissue. The primary hepatocytes were gathered by 500 rpm centrifugalization at 4 °C for 5 min, and repeated the above steps 3 times. The isolated hepatocytes were fostered in William’S Medium E (Cat# GNM41250-2, Genom, China) supplemented with 10% fetal bovine serum (Cat# 10099-141C, Gibco, USA), and 1% penicillin–streptomycin (Cat# 15070-063, Gibco, USA) at 37 °C cell incubator with an atmosphere of 5% CO_2_.

Normal human hepatocytes (L02 cells) and human embryonic kidney (HEK)-293T cells were cultured in Roswell Park Memorial Institute medium (Lot# 6123008, Gibco, USA) supplemented with 10% fetal bovine serum, streptomycin (100 μg/mL), and penicillin (100 U/mL) at 37 °C under an atmosphere of 5% CO_2_/95% air.

To achieve USP13 and TAK1 overexpression, Lipofectamine 3000 reagent (Cat# L3000150, Invitrogen Corporation, Carlsbad, CA, USA) was utilized to transfect L02 cells with USP13 or TAK1 plasmid vector (constructed by Shanghai Genechem Co., Ltd., Shanghai, China) following the instructions from the manufacturer. Moreover, 1 mM (final concentration) free fatty acids [FFAs (2:1 oleate acid–to–palmitate acid ratio)] was used for 24 h to treat L02 cells and 0.5 mM (final concentration) FFA was used for 24 h to treat primary hepatocytes to induce the models of lipid accumulation. Cells treated with bovine serum albumin (BSA) served as the control group.

### Metabolic studies

A glucometer (Abbott Laboratories, Abbott Park, IL, USA) was used to determine the fasting blood glucose level. After fasting for 12–14 h, the mice received an intraperitoneal injection of glucose (1 g/kg) prior to the glucose tolerance test (GTT). The insulin tolerance test (ITT) was performed after a 6-h fast by injecting insulin (0.75 IU/kg) intraperitoneally into mice. Blood glucose was measured 15, 30, 60, 90, and 120 min after the injection. The insulin signaling pathway was evaluated in mice after a 6-h fast. The mice were injected with 1 IU/kg of insulin intraperitoneally 15 min before being sacrificed.

An automated biochemical analyzer (OLYMPUS AU640, Olympus, Japan) was utilized to determine the levels of serum biochemical parameters, including insulin, total cholesterol (TC), fasting glucose, aspartate aminotransferase (AST), triglycerides (TG), and alanine aminotransferase (ALT). The levels of serum inflammatory indicators, including IL-1β, IL-6, and tumor necrosis factor-alpha (TNF-α) were detected using an ELISA kit (IL-1β:Cat# EK201B/3-96; IL-6: Cat# EK206/3-96; TNF-α:Cat# EK282/4-96; Hangzhou Multisciences Biotech, Co., Ltd., Hangzhou, China) or (IL-1β: Cat# 88–7013; IL-6: Cat# 88-7064; Invitrogen Corporation) according to the manufacturer’s instructions. Hepatic and cellular TG content were detected using a TG assay kit (Cat#65336, Abcam plc, Cambridge, England) according to the manufacturer’s instructions. Total protein levels of cells were measured with a bicinchoninic acid (BCA) protein assay kit (Cat# P0010S, Beyotime Institute of Biotechnology, Shanghai, China) and normalized to cellular TG levels. Hepatic TC levels were measured with a cholesterol ester detection kit (Cat# ab65359, Abcam plc). All assays were conducted in accordance with the manufacturers’ protocols.

### Histologic and immunofluorescence analysis

Liver samples were collected from euthanized mice and immediately immersed in 4% paraformaldehyde for fixation. Fixed liver tissues were dehydrated through a graded series of ethanol, cleared in xylene, and embedded in paraffin. Additional liver samples were snap-frozen in liquid nitrogen and stored for Oil Red O staining. Paraffin-embedded liver tissues were sectioned into 5-μm thick slices using a microtome, and frozen liver tissues were sectioned into 10-μm thick slices using a cryostat. For Hematoxylin and Eosin (H&E) staining, paraffin sections were deparaffinized, rehydrated through descending concentrations of ethanol to distilled water, stained with hematoxylin, rinsed in tap water, counterstained with eosin, dehydrated, cleared, and mounted. Cryosections were fixed in 10% formalin for 10 min, rinsed in distilled water, stained with Oil Red O solution for 15 min, washed with 60% isopropanol, and mounted in an aqueous mounting medium. For immunofluorescence staining, the liver sections were deparaffinized with gradients of xylene and alcohol, then incubated with antigen retrieval buffer for 15 min, and probed overnight with primary antibodies at 4 °C followed by secondary antibodies conjugated with fluorescence probes. Finally, the sections were mounted on glass slides with Anti-Fade Fluorescence Mounting Medium (Abcam plc) and the nuclei were stained with 4ʹ,6-diamidino-2-phenylindole for 20 min.

### Cellular oil red O staining

Lipid droplets were observed through cellular Oil Red O staining to demonstrate lipid accumulation. L02 cells were plated on to glass slips of 6-well plates and immersed in FFAs or bovine serum albumin (BSA) for 24 h. The glass slides were then fixed in 4% paraformaldehyde for 30 min, followed by Oil Red O stain for 60 min; hematoxylin was used to stain the nuclei for 1 min.

### RNA preparation and quantitative reverse transcription polymerase chain reaction (RT-qPCR) analysis

TRIzol reagent was supplied by Invitrogen (Cat#15596026, Invitrogen Corporation) and used to isolate total RNA from liver tissue or cells according to the manufacturer’s instructions. PrimeScrip RT Master Mix (Cat# RR036A, Takara Bio, Inc., Shiga, Japan) was used to obtain complementary deoxyribonucleic acid (cDNA) through reverse transcription of RNA (1 µg). The RT-PCR system (Applied Biosystems, Carlsbad, CA, USA) was used to implement RT-qPCR with SYBR Green Premix Ex Taq (Cat# RR420A, Takara). GAPDH expression was used for normalization of relative gene expression and the 2^−ΔΔCt^ method was applied for data analysis. Supplemental Table 1 lists the primers used in qRT-PCR.

### Western blotting assay

The radio-immunoprecipitation assay lysis buffer (Cat# P0013B, Beyotime Institute of Biotechnology), which was added to the phosphatase-protease inhibitor mixture (Cat# P1048, Beyotime Institute of Biotechnology), was used for homogenization of cells and tissue specimens. The BCA protein assay kit (Cat# P0010S, Beyotime Institute of Biotechnology) was used to measure the protein concentration, followed by 10% sodium dodecyl sulfate–polyacrylamide gel electrophoresis (SDS-PAGE) of protein lysates together with polyvinylidene fluoride (PVDF) membrane (EMD Millipore Corporation, Billerica, MA, USA) transfer. The membranes were subsequently cultured at 4 °C overnight with the specified primary antibodies and incubated at room temperature for 1 h with secondary antibodies. Bands were visualized using the electrochemiluminescence reagent (Cat# P90719, EMD Millipore Corporation). ImageJ software (https://imagej.net/ij/) was utilized for quantification of protein bands. The antibodies used in Western blotting are listed in Supplemental Table 2.

### Protein immunoprecipitation and ubiquitination assay

Cell proteins were immunoprecipitated, then 800 µl of immunoprecipitation (IP) lysis buffer (Cat# P0013, Beyotime Institute of Biotechnology) was added to a 10-cm cell dish. The cell dish was placed on ice for 30 min, followed by centrifugation of the cell lysates at 12,000*g* for 15 min at 4 °C. Next, the supernatants were transferred to fresh centrifuge tubes to determine the protein content using a BCA protein assay kit. Protein A/G magnetic beads (Cat# HY-K0202, MedChemExpress, Monmouth Junction, NJ, USA) were used to incubate the specified primary antibodies at 4 °C for 2 h, which were subsequently added to corresponding protein for overnight incubation at 4 °C. Or, the protein samples were incubated at 4 °C overnight with anti-Myc agarose (Cat# M20012, Abmart, Shanghai, China) or anti-FLAG M2 affinity gel (Lot# SLCK8400, Sigma-Aldrich, St. Louis, MO, USA). The immunoprecipitated proteins were washed the next day and mixed with SDS-PAGE loading buffer (Cat# P0010S, Beyotime Institute of Biotechnology), followed by 5 min of heating at 100 °C and a 3-min centrifugation to prepare the protein specimens for Western blotting.

The frozen liver tissues were ground in liquid nitrogen for IP of liver tissue protein and added to 1 ml of IP lysis buffer. Then, the lysates underwent centrifugation at 12,000*g* for 15 min at 4 °C. The following steps referred to above cells.

### RNA sequencing

The samples were the livers of HFD AAV-GFP and HFD AAV-USP13 mice. RNA sequencing was performed by OE Biotechnology Co., Ltd. (Shanghai, China). Total RNA was extracted using the TRIzol reagent (Invitrogen, CA, USA) according to the manufacturer’s protocol. Fold change was used to identify differentially expressed genes, and *P* values were calculated with the t-test. A fold change ≥ 2.0 and a *P* value ≤ 0.05 was set as the threshold for significantly differential expression genes. Kyoto Encyclopedia of Genes and Genomes (KEGG) pathway enrichment analysis and gene set enrichment analysis (GSEA) were conducted to indicate the biological functions and pathways of differential expression genes.

### Statistical analysis

GraphPad software (GraphPad Software, Inc., San Diego, CA, USA) was used for statistical analyses. The mean plus standard error of the mean (SEM) was used to present the study data. An unpaired two-tailed Student’s t-test or ANOVA was used to assess inter-group disparities. A probability (*P*) value < 0.05 was considered statistically significant. Increasing significance levels were defined as a* P* < 0.05, *P* < 0.01, *P* < 0.001 and *P* < 0.0001.

## Results

### Downregulation of USP13 protein in vitro model of NAFLD and in livers of in vivo model of NAFLD

**Fig. 1:**
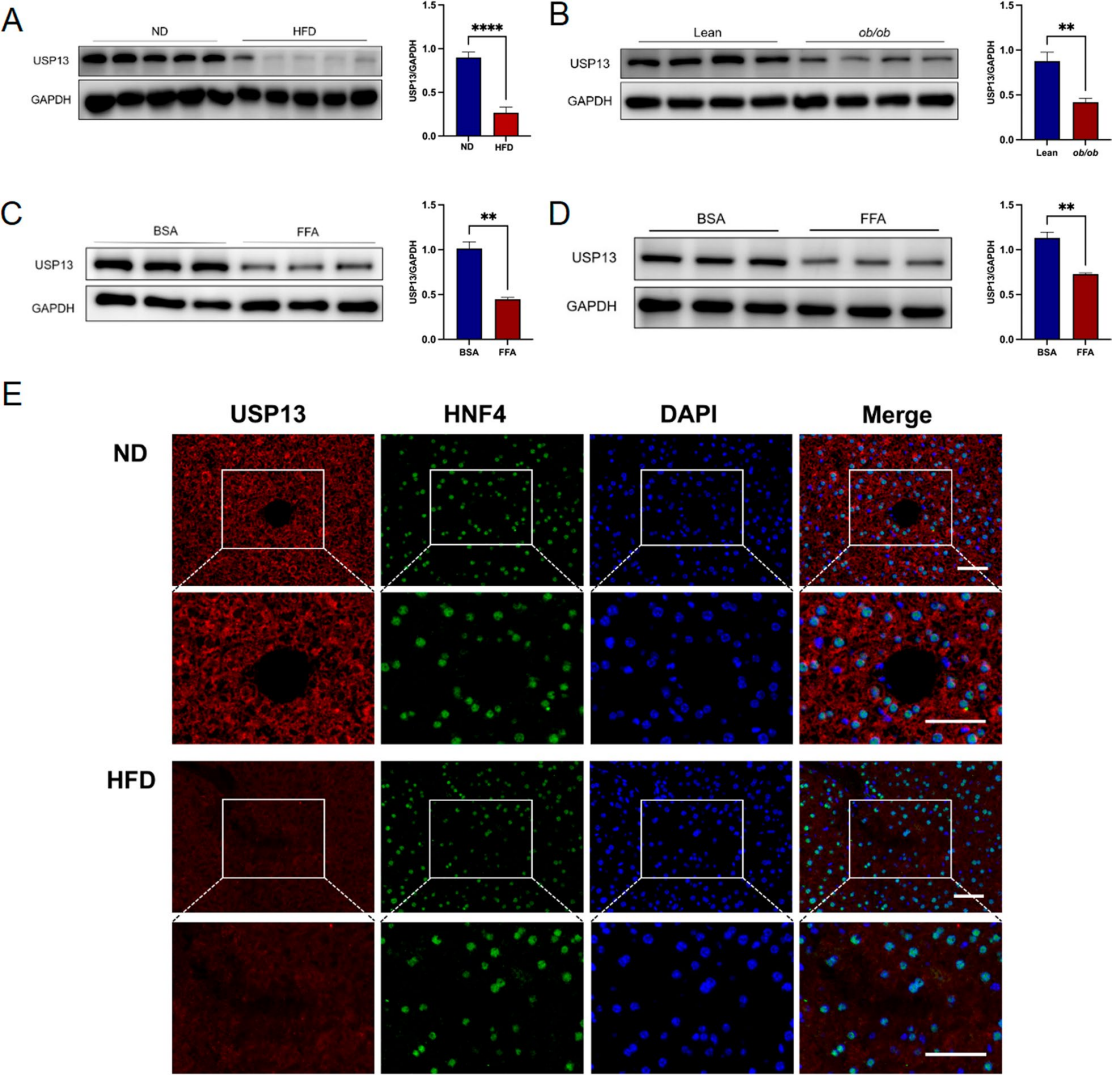
Downregulation of USP13 protein in vitro model of NAFLD and in livers of in vivo model of NAFLD. **A** Western blotting to detect the expression of USP13 in livers of ND-fed or HFD-fed mice (n = 5/group). **B** Western blotting to detect the expression of USP13 in livers of wildtype (Lean) mice or *ob/ob* mice at 16 weeks (n = 4/group). **C** The expression protein levels of USP13 in L02 cells treated with BSA or 1 mM FFA for 24 h (n = 3/group, 3 independent experiments). **D** The expression protein levels of USP13 in mouse primary hepatocytes treated with BSA or 0.5 mM FFA for 24 h (n = 3/group, 3 independent experiments). **E** Immunofluorescence images stained with corresponding antibodies against USP13 (red) to visualize the expression and localization of USP13 in hepatocytes. Nuclei were stained with HNF4 (green) and DAPI (blue; scale bar, 50 μm). Data are presented as mean ± SEM, *P < 0.05, **P < 0.01, ***P < 0.001 and ****P < 0.0001 as determined by Student’s t-test. *NAFLD* nonalcoholic fatty liver disease, *DAPI* 4',6-diamidino-2-phenylindole, *GAPDH* glyceraldehyde 3-phosphate dehydrogenase, *HNF4* hepatocyte nuclear factor 4

We first established the HFD-induced NAFLD model in mice, and the differences of fasting body weight and fasting blood glucose between ND-fed mice and HFD-fed mice were significant (Supplementary Fig. 1A and B). The liver appearance, H&E and Oil Red O staining of liver sections in ND-fed mice and HFD-fed mice showed severe hepatic steatosis and inflammation in the livers of HFD-fed mice (Supplementary Fig. 1C), which indicated that the mouse NAFLD model was successfully established. To investigate the role of USP13 in NAFLD, we first assessed the expression levels of USP13 in models of NAFLD. The immunohistochemistry indicated that USP13 expression was decreased in the liver of HFD-fed mice compared with ND-fed mice (Supplementary Fig. 1D). HFD-fed mice had reduced protein expression of USP13 compared to ND-fed mice based on Western blot analysis of liver samples (Fig. 1A). Similar results were obtained in *ob/ ob* mice (Fig. 1B). Additionally, the level of USP13 expression was also decreased in L02 cells and mouse primary hepatocytes after a 24-h treatment with 1 mM or 0.5 mM FFAs (Fig. 1C and D). Moreover, immunofluorescence co-localization of USP13 and hepatocyte nuclear factor 4 indicated that HFD-fed mice had a lower level of USP13 expression in hepatic tissue sections, mainly in the hepatocytes (Fig. 1E). The reduced expression of USP13 suggested its potential role in NAFLD.

### USP13 restrains lipid accumulation and inflammatory response in L02 hepatocytes following metabolic stimulation

**Fig. 2:**
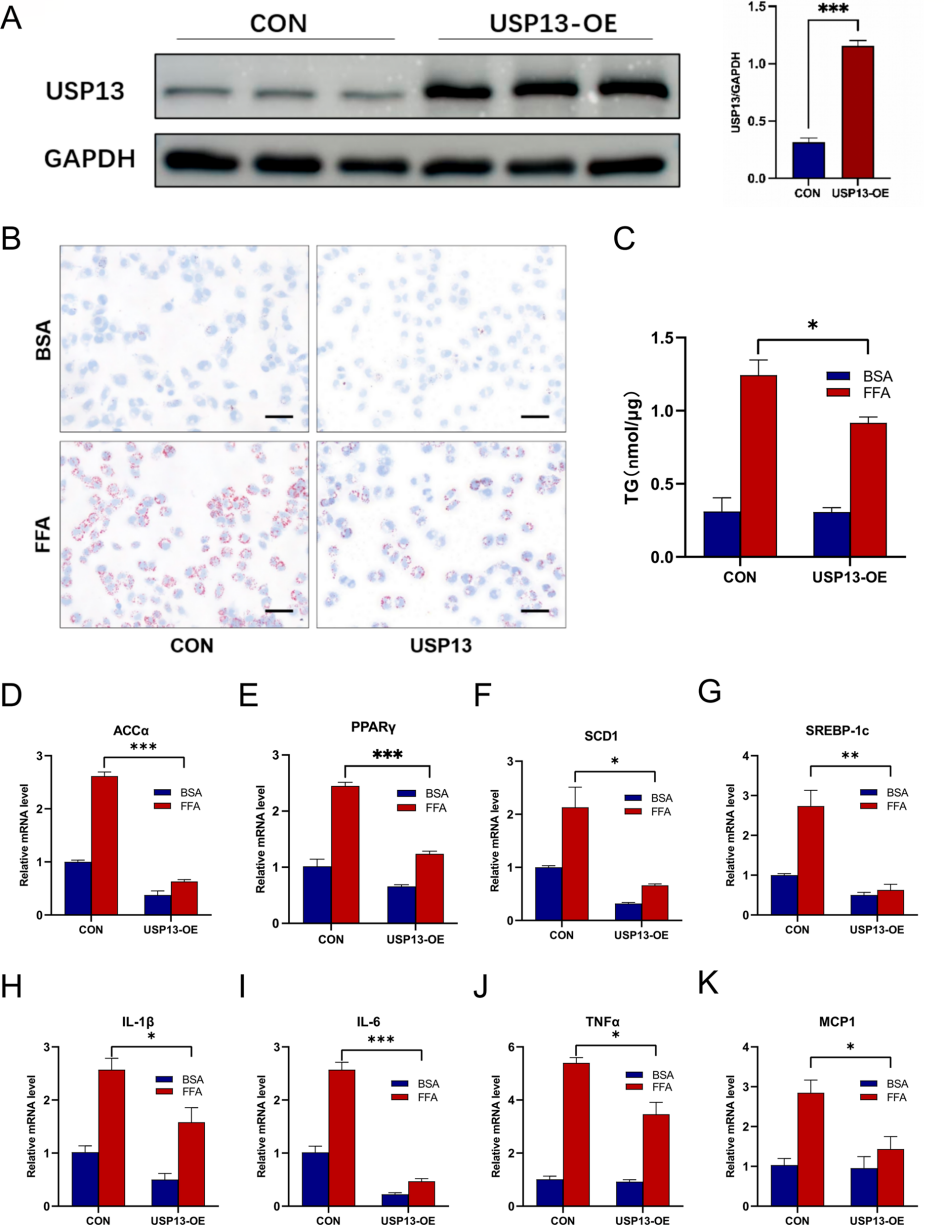
Overexpression of USP13 prevented lipid accumulation and inflammatory response in L02 cells after FFAs stimulation. **A** Western blot analysis of USP13 expression levels in L02 cells transfected with Flag-tagged USP13. **B** Representative Oil Red O staining of L02 cells in the indicated groups following BSA or FFA stimulation for 24 h (scale bar, 50 μm). **C** Cellular contents of TG in USP13-overexpressing and control L02 cells cultured with BSA or 1 mM FFA for 24 h (n = 3/group). **D**–**K** mRNA expression of relative genes including fatty acid synthesis and proinflammatory factors in L02 hepatocytes treated with BSA or FFA from the indicated groups (n = 3/group). All in vitro experiments were performed 3 independent times. All data are shown as the mean ± SEM. *P < 0.05, **P < 0.01, ***P < 0.001 as determined by Student’s t-test

Next, we overexpressed USP13 in the L02 cell line by transfection with USP13 plasmids to elucidate its function in hepatocytes (Fig. 2A). As revealed by Oil Red O staining and TG quantification, FFAs-induced lipid accumulation was obviously inhibited by USP13 overexpression (Fig. 2B and C). Genes, such as acetyl coenzyme A carboxylase α (ACCα), peroxisome proliferator–activated receptor gamma (PPARγ), sterol regulatory element-binding protein-1c (SREBP-1c) and stearoyl-CoA desaturase 1 (SCD1), had significantly decreased levels of mRNA expression in FFA-treated L02 cells with USP13 overexpression (Fig. 2D–G). In addition, inflammatory genes including IL-1β, IL-6, TNFα and MCP1 were also restrained in USP13 overexpression L02 cells treated with FFA (Fig. 2H–K). Therefore, hepatocyte USP13 alleviates lipid accumulation as well as inflammation in L02 cells subjected to FFAs stimulation.

### USP13 overexpression ameliorates hepatic steatosis, insulin resistance and inflammation

**Fig. 3:**
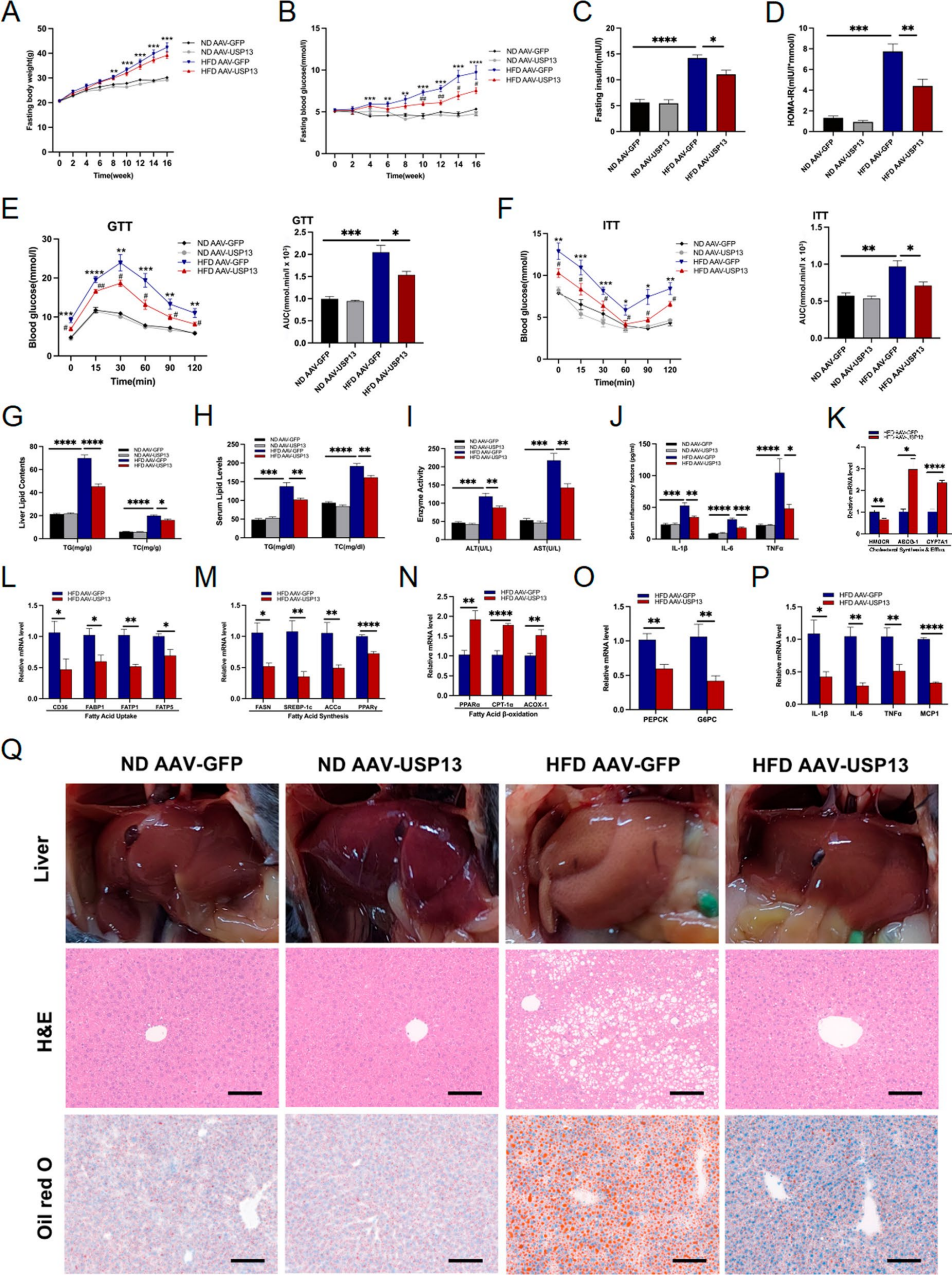
Hepatic USP13 overexpression attenuates insulin resistance, hepatic steatosis and inflammation. **A**, **B** Fasting body weights and blood glucose levels of ND or HFD feeding 0–16 weeks in mice administered with the AAV-GFP or AAV-USP13 (n = 6/group), respectively. **C**, **D** Fasting insulin levels and HOMA-IR indexes of ND or HFD fed for 16 weeks in mice administered with the AAV-GFP or AAV-USP13, respectively (n = 6/group). **E**, **F** GTTs and ITTs, and the corresponding AUC for GTTs and ITTs of ND or HFD fed for 16 weeks in mice administered with the AAV-GFP or AAV-USP13(n = 6/group), respectively. **G**, **H** Hepatic contents and serum levels of TG, TC in the indicated groups (n = 6/group). **I** Serum levels of ALT and AST in the indicated groups (n = 6/group). **J** Serum levels of inflammatory factors in the indicated groups (n = 6/group). **K**–**P** RT-PCR to detect mRNA levels of genes related to cholesterol synthesis and efflux, fatty acid uptake, fatty acid synthesis, fatty acid β-oxidation, gluconeogenesis and inflammation in liver samples of HFD fed for 16 weeks in mice administered with the AAV-GFP or AAV-USP13 (n = 6/group). **Q** Liver appearance, H&E and Oil Red O staining of liver sections in ND or HFD fed mice administered with the AAV-GFP or AAV-USP13 (scale bar, 100 μm). The data are presented as the means ± SEM. For **A**, **B**, **E**, **F**, *P < 0.05 versus ND AAV-GFP, **P < 0.01 versus ND AAV-GFP, ***P < 0.001versus ND AAV-GFP, ****P < 0.0001versus ND AAV-GFP; ^#^*P* < 0.05 versus HFD AAV-GFP, ^##^*P* < 0.01 versus HFD AAV-GFP, ^###^*P* < 0.001 versus HFD AAV-GFP, ^####^*P* < 0.0001 versus HFD AAV-GFP. *HOMA-IR* homeostasis model assessment of insulin resistance, *AUC* area under the curve

To further explore the role of USP13 in NAFLD in vivo, we overexpressed hepatic USP13 in mice via injection of AAV-USP13. HFD or ND was provided to the mice for 16 weeks. The USP13 expression levels in HFD AAV-GFP and HFD AAV-USP13 mice are present in Supplementary Fig. 2A. Compared to HFD AAV-GFP mice, the body weights of HFD AAV-USP13 mice were not significantly different (Fig. 3A). In contrast, the fasting blood glucose levels in USP13 overexpression mice were significantly reduced after being fed HFD for 16 weeks when compared with the control group (Fig. 3B). Additionally, HFD AAV-USP13 mice had a reduced serum insulin levels and homeostasis model assessment of insulin resistance (HOMA-IR) compared to HFD AAV-GFP mice (Fig. 3C and D). Moreover, as shown by ITT plus GTT in mice administered HFD, USP13 overexpression lowered the blood glucose level and improved insulin sensitivity (Fig. 3E and F). In addition, HFD AAV-USP13 mice had attenuated TC and TG content in the liver (Fig. 3G), as well as reduced serum TG and TC levels (Fig. 3H). Moreover, the HFD AAV-USP13 mice proved to have lower serum AST and ALT levels, and the levels of serum inflammatory factors were also decreased (Fig. 3I, J). Reduced lipid accumulation was observed from the hepatic sections of HFD AAV-USP13 mice based on the findings of Oil Red O and H&E staining (Fig. 3Q), which further verified the above results.

The genes related to the synthesis of cholesterol and fatty acid, such as ACCα, HMGCR, FASN, SREBP-1c, and PPARγ, as well as the uptake of fatty acids (FATP1, FABP1, FATP5, and CD36) were manifest as decreased mRNA expression in HFD-fed AAV-USP13 mice. HFD-fed AAV-USP13 mice had significantly increased levels of gene mRNA in cholesterol efflux, including CYP7A1 and ABCG-1, and fatty acid β-oxidation (e.g., CPT-1α, ACOX-1, and PPARα). The levels of G6PC and PEPCK mRNA expression, which are two crucial gluconeogenesis-regulating enzymes, were reduced in hepatic samples of HFD AAV-USP13 mice compared to controls. In agreement with the above results, the IL-6, MCP1, IL-1β, and TNFα mRNA levels were significantly reduced in hepatic samples of HFD AAV-USP13 mice compared to controls. The mRNA expression was present in Fig. 3K–P. All above results further demonstrated the protective role of USP13 against hepatic steatosis, liver impairment and inflammation.

### USP13 deletion exacerbates insulin resistance, hepatic steatosis, and inflammation

**Fig. 4:**
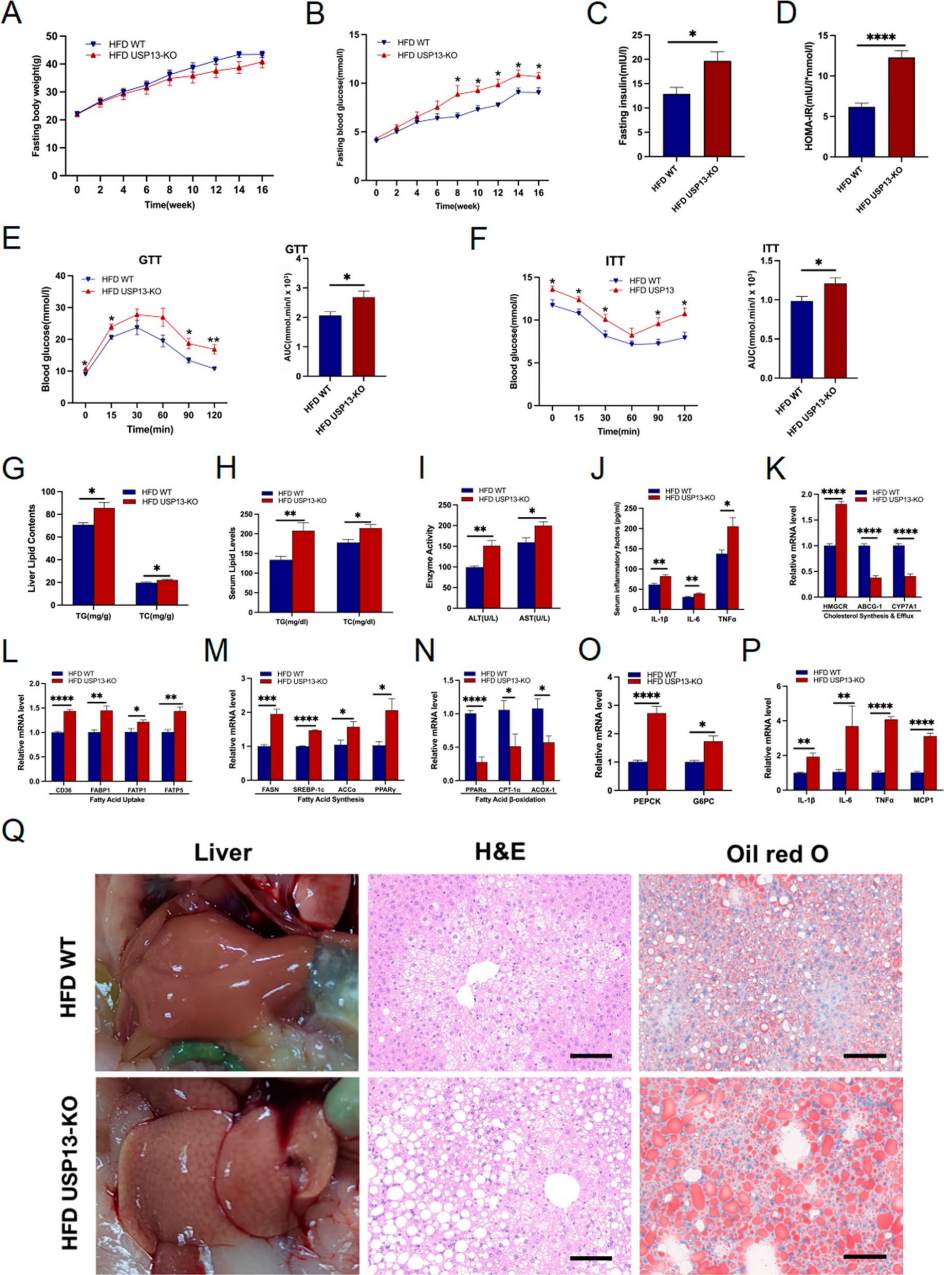
USP13 deficiency aggravates HFD-induced hepatic steatosis, insulin resistance and inflammatory responses. **A**, **B** Fasting body weights and blood glucose levels of HFD feeding 0–16 weeks in WT or USP13-KO mice (n = 6/group), respectively. **C**, **D** Fasting insulin levels and HOMA-IR indexes of HFD fed for 16 weeks in WT or USP13-KO mice (n = 6/group), respectively. **E**, **F** GTTs and ITTs, and the corresponding AUC for GTTs and ITTs (n = 6/group) of HFD fed for 16 weeks in WT or USP13-KO mice, respectively. **G**, **H** Hepatic contents and serum levels of TG, TC in the indicated groups (n = 6/group). **I** Serum levels of ALT and AST in the indicated groups (n = 6/group). **J** Serum levels of inflammatory factors in the indicated groups (n = 6/group). **K**–**P** RT-PCR to detect mRNA levels of genes related to cholesterol synthesis and efflux, fatty acid uptake, fatty acid synthesis, fatty acid β-oxidation, gluconeogenesis and inflammation in liver samples of HFD fed for 16 weeks in WT or USP13-KO mice (n = 6/group). **Q** Liver appearance, H&E and Oil Red O staining of liver sections in WT or USP13-KO mice with HFD feeding for 16 weeks (scale bar, 100 μm). The data are presented as the means ± SEM.*P < 0.05, **P < 0.01, ***P < 0.001, ****P < 0.0001 as determined by Student’s t-test. For **A**, **B**, **E**, **F**, *P < 0.05 versus HFD WT and **P < 0.01 versus HFD WT. *HOMA-IR* homeostasis model assessment of insulin resistance, *AUC* area under the curve

To further confirm the role of USP13 in NAFLD, we generated USP13-KO mice and were fed HFD for 16 weeks. The USP13 expression levels in HFD WT and HFD USP13-KO mice are present in Supplementary Fig. 2B. The body weights were similar between the USP13-KO mice and the control group (Fig. 4A), whereas HFD USP13-KO mice exhibited higher fasting concentrations of glucose, insulin levels and HOMA-IR values than the controls (Fig. 4B–D). GTT and ITT showed that USP13 deficiency impaired glucose tolerance and insulin sensitivity after HFD feeding (Fig. 4E and F). Also, in USP13-KO mice fed the HFD, hepatic and serum levels of TC and TG were relatively increased (Fig. 4G and H), along with elevated serum ALT, AST and inflammatory factors (Fig. 4I, J). Moreover, the hepatic sections of USP13-KO mice exhibited increased lipid accumulation and inflammation under Oil Red O in combination with H&E staining (Fig. 4Q), which suggests the deficiency of USP13 aggravate liver steatosis and inflammation.

The mRNA levels of genes associated with the uptake of fatty acids (CD36, FABP1, FATP1, FATP5), synthesis of fatty acids and cholesterol (ACCα, HMGCR, FASN, SREBP-1c, PPARγ), enzymes that regulate gluconeogenesis (PEPCK, G6PC) and inflammatory factors (IL-6, MCP1, IL-1β, and TNFα) were increased, while the mRNA levels of genes associated with fatty acid β-oxidation (CPT-1α, ACOX-1, PPARα) and cholesterol efflux (CYP7A1, ABCG-1) were markedly reduced in the liver tissues of USP13-KO mice fed the HFD (Fig. 4K–P). These results were manifest as aggravated inflammation, insulin resistance, and liver steatosis of USP13-KO after HFD stimulation.

### USP13 regulates inflammatory and insulin signaling pathways in NAFLD

**Fig. 5:**
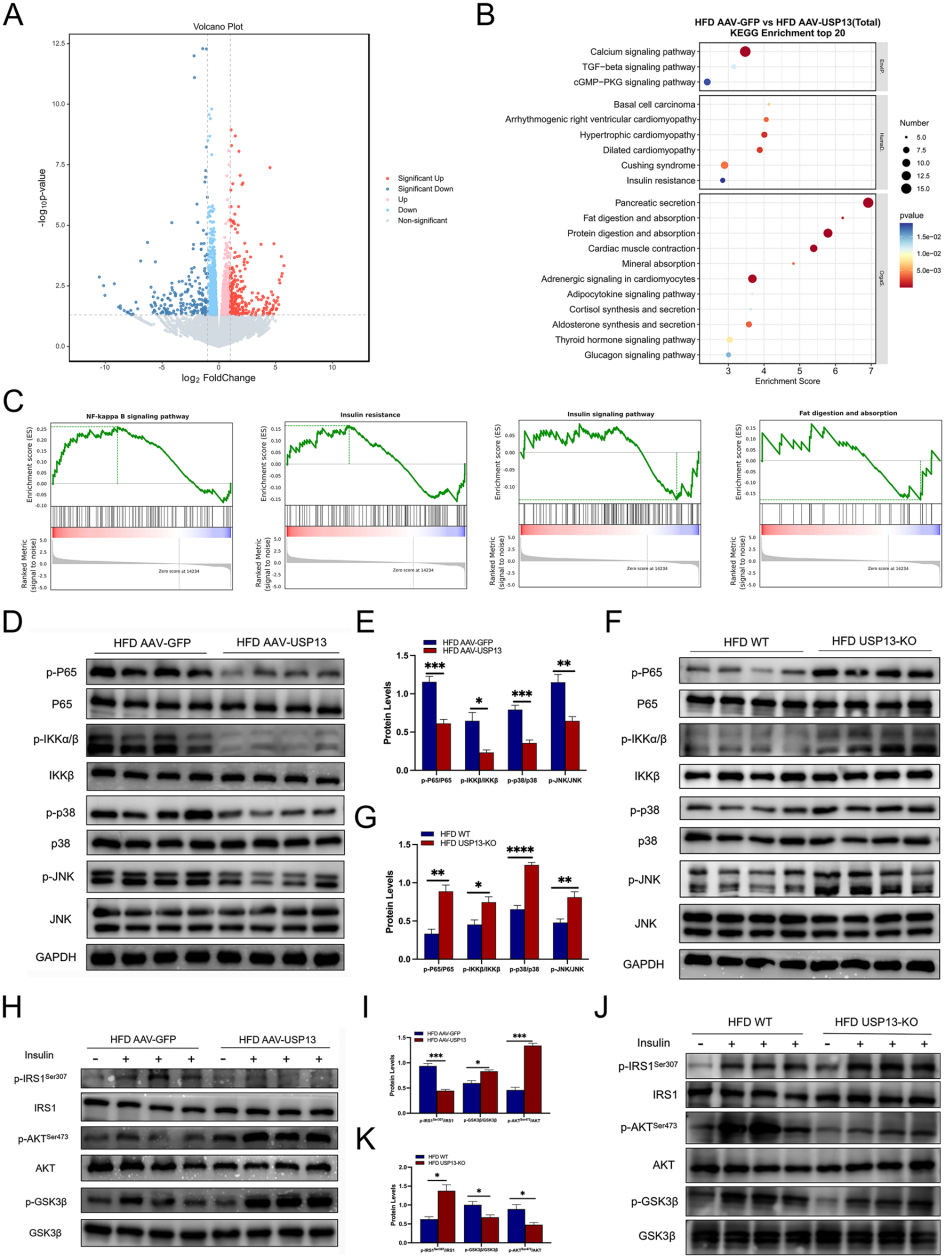
USP13 regulates inflammatory and insulin signaling pathways in NAFLD. **A** Volcano map of differential gene expression of USP13 overexpression in liver tissue of HFD feeding mice administered with the AAV-GFP or AAV-USP13. **B** Kyoto Encyclopedia of Genes and Genomes (KEGG) pathway enrichment analysis of differentially expressed genes presented as a bubble chart. **C** Gene set enrichment analysis of genes expression in liver tissue: NF-κB, insulin, insulin resistance and fat digestion and absorption signaling pathways. **D**, **E** Phosphorylation levels of JNK, p38, and NF-κB signaling pathway in liver samples collected from HFD feeding mice administered with the AAV-GFP or AAV-USP13 (n = 4/group), and phosphorylated protein levels were normalized to total protein. **F**, **G** Phosphorylation levels of JNK, p38, and NF-κB signaling pathway in liver samples collected from HFD feeding 16 weeks in WT or USP13-KO mice (n = 4/group), and phosphorylated protein levels were normalized to total protein. **H**, **I** The key players’ phosphorylated levels of insulin signaling pathway in the liver samples collected from HFD feeding mice administered with the AAV-GFP or AAV-USP13 (n = 4/group), and phosphorylated protein levels were normalized to total protein. **J**, **K** The key players’ phosphorylated levels of insulin signaling pathway in the liver samples collected from HFD feeding 16 weeks in WT or USP13-KO mice (n = 4/group), and phosphorylated protein levels were normalized to total protein. The data are presented as the means ± SEM. *P < 0.05, **P < 0.01, ***P < 0.001, ****P < 0.0001 as determined by Student’s t-test

To further explored the underlying mechanisms of USP13 in NAFLD, RNA sequencing of livers in HFD AAV-GFP and HFD AAV-USP13 mice was conducted, and subsequent KEGG pathway enrichment analysis and GSEA were performed. The results of the volcano plot indicated that 273 genes were up-regulated and 206 genes were down-regulated after hepatic overexpression of USP13 (Fig. 5A). KEGG pathway enrichment analysis suggested a significant role of USP13 in insulin resistance signaling pathways and fat digestion and absorption signaling pathways (Fig. 5B). In addition, GSEA indicated NF-κB and insulin resistance signaling pathways were inhibited in HFD AAV-USP13 group, while insulin signaling pathways, and fat digestion and absorption signaling pathways were facilitated in HFD AAV-USP13 group (Fig. 5C).

Then, we further examined these signaling pathways by Western blot. With respect to the MAPK and NF-κB signaling pathways, IKKβ, P65, JNK and p38 phosphorylation were significantly inhibited in livers of HFD AAV-USP13 mice compared to livers of HFD AAV-GFP mice (Fig. 5D and E). Conversely, phosphorylation levels of the JNK, p38, and NF-κB pathways was significantly upregulated in USP13-KO mice fed the HFD as compared with the controls (Fig. 5F and G).

Prompted by the GSEA results that insulin resistance were inhibited in HFD AAV-USP13 group, while insulin signaling pathways were facilitated in HFD AAV-USP13 group, thus, phosphorylation levels of the insulin receptor substrate 1 (IRS1)-protein kinase B (AKT)-glycogen synthase kinase-3 (GSK3β) axis was examined. Serine (Ser) 473-phosphorylated AKT, and phosphorylated GSK3β were triggered, while the level of Ser307-phosphorylated IRS1 was decreased in hepatic sections from HFD AAV-USP13 mice (Fig. 5H and I). Reduced IRS1 phosphorylation (Ser307) and increased AKT phosphorylation (Ser473) revealed enhanced insulin sensitivity of HFD AAV-USP13 mice. We further examined the insulin signaling pathway in livers of HFD WT and HFD USP13-KO mice(Fig. 5J and K), and increased IRS1 phosphorylation (Ser307), decreased AKT phosphorylation (Ser473) and GSK3β phosphorylation were found in the livers of HFD USP13-KO mice. These phenotype alterations indicated the role of USP13 in attenuating inflammation, insulin resistance, and liver steatosis in NAFLD.

### USP13 interacts with TAK1, and represses TAK1 activation though removing ubiquitination

**Fig. 6:**
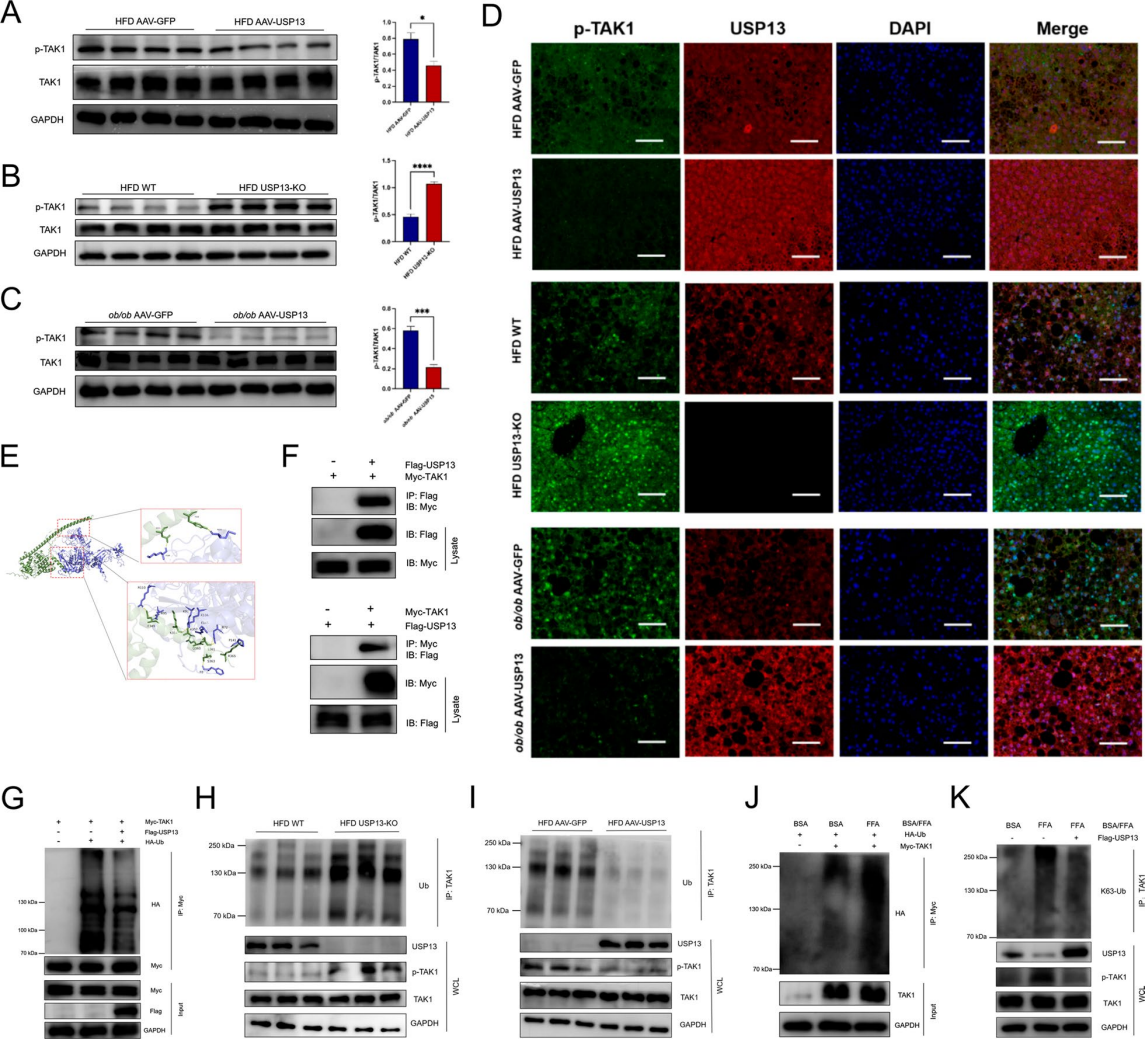
USP13 inhibits the activation of TAK1 by removing ubiquitination. **A**–**C** Phosphorylated TAK1 expression levels in the livers of 16 weeks HFD feeding mice administrated with AAV-USP13 (**A**), USP13-KO (**B**) mice with HFD diet and (**C**) *ob/ob* AAV-USP13 (n = 4/group), and the AAV-GFP, WT and *ob/ob* AAV-GFP mice were used as controls. Expression levels were normalized by phosphorylated to total TAK1 (graphs in the right panel). **D** Representative immunofluorescence images of USP13 and phosphorylated TAK1 co-expression in mice liver sections of the indicated groups (scale bar, 100 μm). **E** Schematic diagram of USP13 and TAK1 molecular docking. **F** Interaction between USP13 and TAK1 detected by coimmunoprecipitation in 293T cells co-transfected with Flag-tagged USP13 and Myc-tagged TAK1. **G** After co-transfecting Flag-tagged USP13, Myc-TAK1 and HA-Ub plasmids into 293T cells, TAK1 ubiquitination was analyzed by immunoprecipitation and western blotting analyses. **H**, **I** Ubiquitination of TAK1 in the liver samples obtained from AAV-USP13 or USP13-KO mice compared with the controls was detected by immunoprecipitation with anti-TAK1 antibodies, followed by western blot with anti-Ub antibody. **J** After co-transfecting Myc-TAK1 and HA-Ub into L02 cells treated with BSA or FFA, TAK1 ubiquitination was analyzed by immunoprecipitation with anti-Myc antibodies, followed by western blot with anti-HA antibody. **K** With or without transfecting Flag-tagged USP13 plasmids into L02 cells, K63-linked polyubiquitination of TAK1 in L02 cells treated with BSA or FFA was detected by immunoprecipitation with anti-TAK1 antibodies, followed by western blot with anti-K63-Ub antibody. For **A**–**C**, *P < 0.05, **P < 0.01, ***P < 0.001, ****P < 0.0001 versus control group. *GAPDH* glyceraldehyde 3-phosphate dehydrogenase, *IB* immunoblot, *IP* immunoprecipitation, *WCL* whole-cell lysate

In light of the above findings that USP13 inhibits the MAPK and NF-κB signaling pathways, we considered TAK1, a known upstream kinase of these pathways, as a potential downstream target of USP13. To assess the impact of USP13 on TAK1, we measured TAK1 levels in the livers of various mouse models. As shown in Fig. 6A–C, while the total TAK1 protein level remained unchanged, TAK1 phosphorylation was markedly suppressed by AAV-USP13 in HFD-fed and *ob/ob* mice. Conversely, HFD-fed USP13-KO mice exhibited increased TAK1 phosphorylation levels compared to controls. Immunofluorescence analysis further confirmed that TAK1 activation was significantly inhibited in HFD-fed AAV-USP13 and *ob/ob* AAV-USP13 mice, but notably augmented in HFD-fed USP13-KO mice (Fig. 6D).

We then investigated whether USP13 physically interacts with TAK1. Using molecular docking and co-immunoprecipitation techniques, we demonstrated that USP13 binds to TAK1 (Fig. 6E and F). Given the established role of TAK1 ubiquitination in its activation, we hypothesized that USP13 inhibits TAK1 activation by reducing its ubiquitination. To test this, we co-transfected 293T cells with Myc-TAK1, Flag-USP13, and HA-Ub. Overexpression of USP13 resulted in decreased TAK1 ubiquitination (Fig. 6G). To further validate our hypothesis, we examined the ubiquitination and phosphorylation levels of TAK1 in hepatic tissues of HFD-fed USP13-KO and AAV-USP13 mice. The livers of USP13-KO mice showed enhanced TAK1 ubiquitination and phosphorylation, while USP13 overexpression led to decreased levels of both (Fig. 6H and I).

Furthermore, TAK1 ubiquitination was increased in FFA-treated L02 cells compared to BSA-treated controls (Fig. 6J). Since TAK1 autophosphorylation requires K63-linked polyubiquitination for full activation, we analyzed the effect of USP13 on TAK1 K63-ubiquitination in L02 cells. Compared to BSA-treated controls, FFA-treated L02 cells exhibited increased K63-linked ubiquitination and phosphorylation of TAK1, which were reduced upon USP13 overexpression (Fig. 6K). These findings suggest that USP13 removes K63-linked polyubiquitin chains from TAK1, thereby inhibiting its phosphorylation and activation in L02 cells.

### TAK1 is necessary for USP13 to alleviate NAFLD

**Fig. 7:**
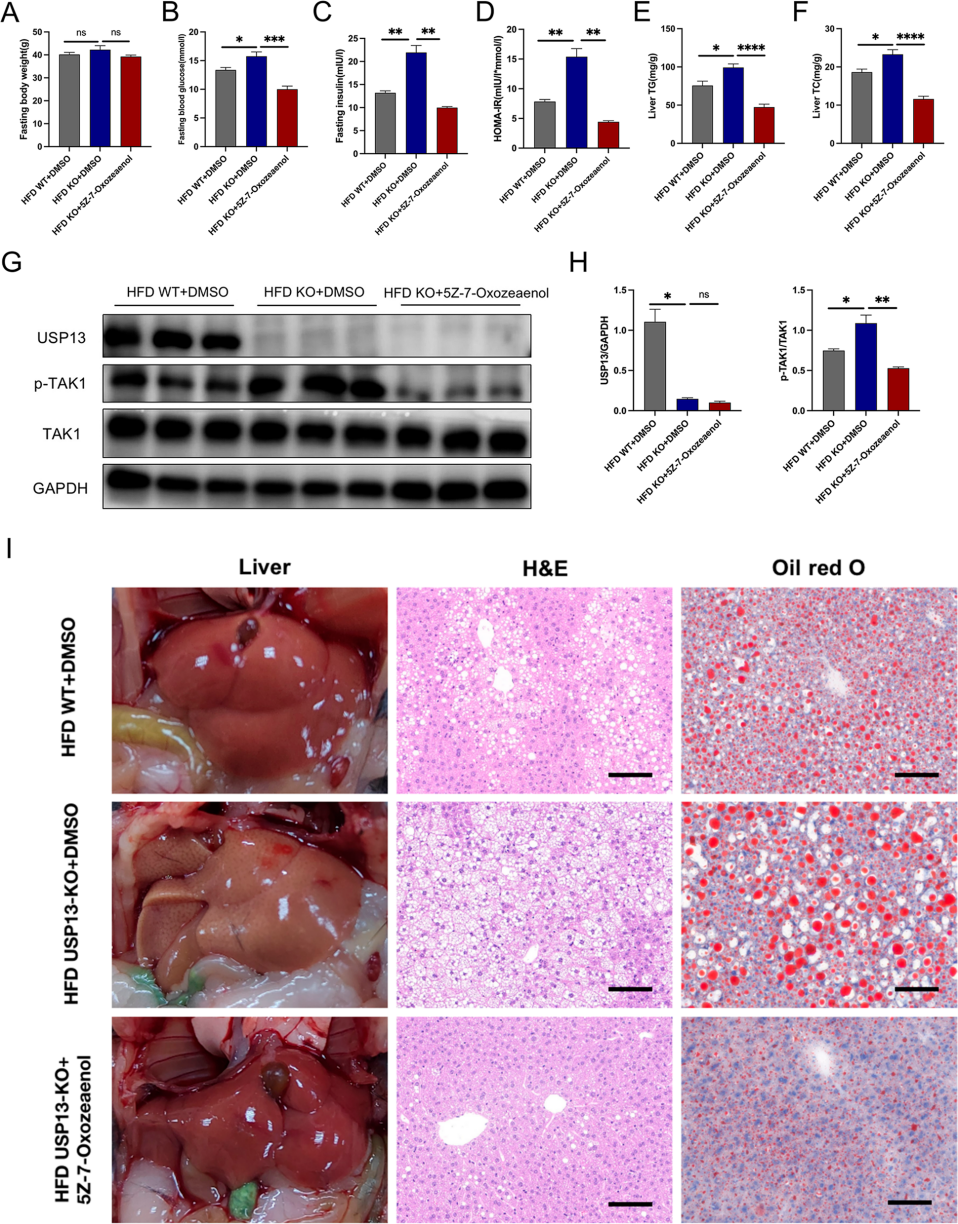
TAK1 is necessary for USP13 to alleviate NAFLD. **A**–**D** Body weights, fasting glucose levels, fasting insulin levels and HOMA-IR indexes of mice in the indicated groups (n = 5/group). **E**–**F** Hepatic TG and TC contents of mice in the indicated groups after 16 weeks of HFD feeding (n = 5/group). **G**, **H** Expression of USP13, phosphorylated and total TAK1 was analyzed in the livers of HFD -fed WT mice, USP13-KO mice treated with DMSO or 5Z-7-Oxozeaenol for 8 weeks, phosphorylated protein levels were normalized to total protein (n = 3/group). **I** Liver appearance, H&E and Oil Red O staining of liver sections of HFD -fed WT mice, USP13-KO mice treated with DMSO or 5Z-7-Oxozeaenol for 8 weeks (scale bar, 100 μm). The data are presented as the means ± SEM. *P < 0.05, **P < 0.01, ***P < 0.001, ****P < 0.0001 as determined by Student’s t-test. *GAPDH* glyceraldehyde 3-phosphate dehydrogenase, *DMSO* dimethyl sulfoxide

In order to verify the necessity of TAK1 in the role of USP13 in NAFLD, we adopted the TAK1 inhibtor-5Z-7-Oxozeaenal to inhibit the activation of TAK1. 8-week HFD-fed USP13-KO mice were received an intraperitoneal injection of 5Z-7-Oxozeaenol, and the other two groups (HFD WT and HFD USP13-KO) were given to the same dimethyl sulfoxide (DMSO). The verification results of USP13 expression and TAK1 activation were present in Fig. 7G and H. We observed that though the body weights of HFD WT + DMSO, HFD KO + DMSO and HFD KO + Oxozeaenol groups were similar (Fig. 7A), the glucose and lipid metabolism disorders were alleviated when suppressing the TAK1 activation in USP13-KO mice (Fig. 7B–F). The results of Oil Red O and H&E staining further verified the effects of inhibiting TAK1, including reduced hepatic lipid accumulation and inflammation (Fig. 7I). Therefore, inhibiting the TAK1 activation could reverse the exacerbation of NAFLD brought from the deficiency of USP13, indicating that targeting TAK1 is necessary for USP13 to alleviate NAFLD.

### Therapeutic role of USP13 in NAFLD in *ob/ob* mice

**Fig. 8:**
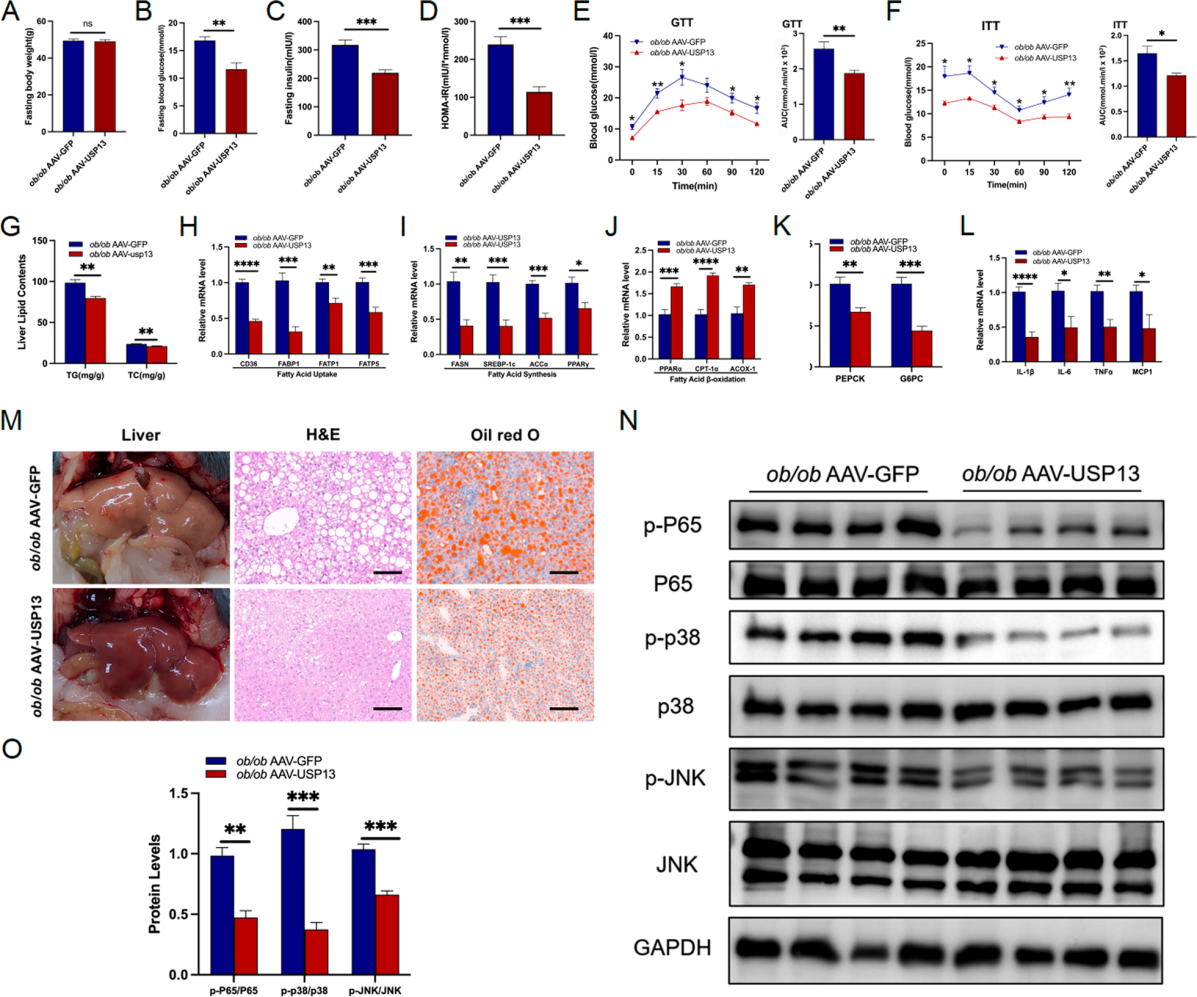
Therapeutic role of USP13 in NAFLD in *ob/ob* mice. **A**–**D** Body weights, fasting glucose levels, fasting insulin levels and HOMA-IR indexes of *ob/ob* mice administered with the AAV-GFP or AAV-USP13 after 4 weeks (n = 6/group). **E**–**F** GTTs and ITTs and the corresponding AUC for GTTs and ITTs in *ob/ob* mice administered with the AAV-GFP or AAV-USP13 after 4 weeks (n = 6/group). **G** Hepatic contents of TG, TC in *ob/ob* mice administered with the AAV-GFP or AAV-USP13 after 4 weeks (n = 6/group). **H**–**L** RT-PCR to detect mRNA levels of genes related to fatty acid uptake, fatty acid synthesis, fatty acid β-oxidation, gluconeogenesis and inflammation in liver samples of *ob/ob* mice administered with the AAV-GFP or AAV-USP13 after 4 weeks (n = 6/group). **M** Liver appearance, H&E and Oil Red O staining of liver sections in *ob/ob* mice administered with the AAV-GFP or AAV-USP13 after 4 weeks (scale bar, 100 μm). **N**–**O** Phosphorylated levels of JNK, p38 and P65 in liver samples of *ob/ob* mice administered with the AAV-GFP or AAV-USP13 after 4 weeks (n = 4/group), and phosphorylated protein levels were normalized to total protein. The data are presented as the means ± SEM. *P < 0.05, **P < 0.01, ***P < 0.001, ****P < 0.0001 as determined by Student’s t-test. *HOMA-IR* homeostasis model assessment of insulin resistance, *AUC* area under the curve

To further verify the therapeutic effects of USP13, 8-week-old *ob/ob* mice were injected with AAV-USP13 (*ob/ob* AAV-USP13) to overexpress hepatic USP13, while the control group were administered AAV-GFP. The USP13 expression levels in *ob/ob* AAV-GFP and *ob/ob* AAV-USP13 mice are present in Supplementary Fig. 2C. Although the two groups had similar body weights (Fig. 8A), the *ob/ob* AAV-USP13 group exhibited reduced HOMA-IR values and insulin levels, along with decreased serum fasting glucose levels (Fig. 8B–D). The GTT and ITT results indicated improved glucose homeostasis and insulin sensitivity in *ob/ob* AAV-USP13 group (Fig. 8E and F). The *ob/ob* AAV-USP13 group had alleviated lipid droplets and inflammation, as demonstrated by Oil Red O and H&E staining (Fig. 8M). Moreover, the livers of *ob/ob* AAV-USP13 mice had reduced TG and TC contents (Fig. 8G), along with lower mRNA expression levels of genes associated with fatty acids synthesis and uptake, gluconeogenesis and inflammation, conversely, the mRNA expression of genes involved in fatty acid β-oxidation was higher in the livers of *ob/ob* AAV-USP13 mice when compared with the controls (Fig. 8H–L). Moreover, the phosphorylation levels of NF-κB, JNK and p38 signaling pathways were significantly inhibited in *ob/ob* AAV-USP13 mice compared to controls (Fig. 8N and O), suggesting a reduction in liver inflammation. To summarize, USP13 suppressed liver steatosis, inflammation, and insulin resistance to demonstrate efficacy in treating *ob/ob* mice.

## Discussion

**Fig. 9:**
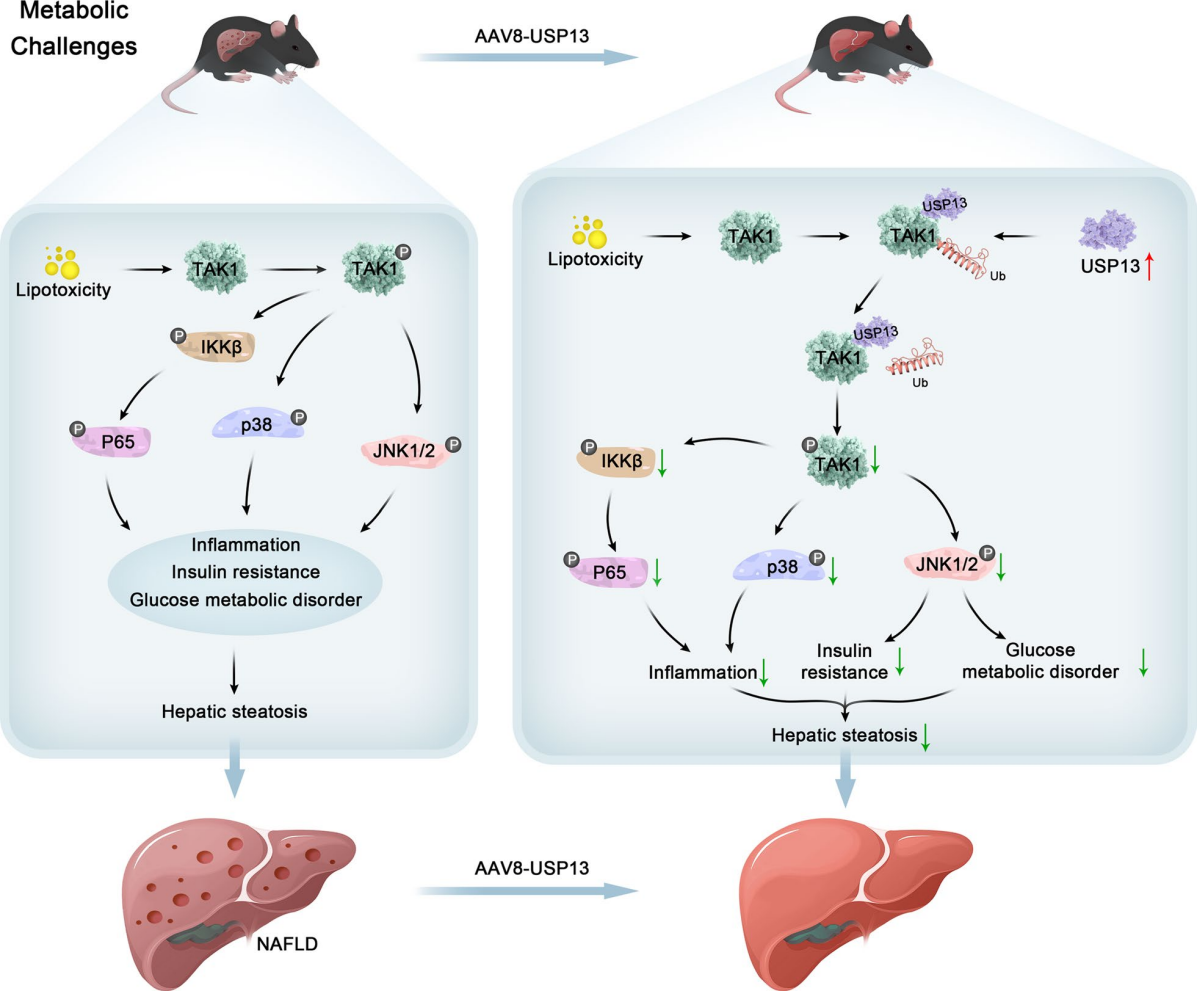
The role of USP13 in NAFLD and its possible molecular mechanisms

As the most frequently-occurring hepatopathy, 20%–27% of patients with NAFLD develop NASH [4], which can progress to liver cirrhosis and ultimately carcinoma [34]. However, NAFLD cannot be effectively treated by medications at this time. Despite the number of studies with a focus on NAFLD, the molecular mechanism underlying NAFLD remains elusive. USP13 expression was shown to be downregulated in response to the treatment with FFA in hepatocytes. USP13 overexpression by injecting with AAV8-USP13 via tail vein mitigated liver steatosis, inflammation, and insulin resistance in *ob/ob* and HFD-fed mice. Moreover, USP13 deficiency achieved the opposite effects. USP13 interacts with TAK1, inhibits TAK1 activation by removing ubiquitination of TAK1, and subsequently inhibits the NF-κB & MAPK signaling pathway activation (Fig. 9), thereby treating NAFLD as a latent molecular target.

As a DUB enzyme, USP13 regulates the de-ubiquitination of multiple substrate proteins to participate in many cellular processes, including mitochondrial energy metabolism, DNA damage, autophagy, and endoplasmic reticulum-associated degradation [35]. The number of studies related to lipid metabolism disorders related to USP13 is limited. In the current study, we further studied the role of USP13 in NAFLD using USP13 overexpression and knockout techniques after demonstrating reduced USP13 in obese mice and FFA-treated L02 cells. Liver steatosis, insulin resistance and inflammation were alleviated by hepatic USP13 overexpression in obese mice, and aggravated in USP13 knockout HFD-induced mice. Therefore, USP13 is an important protective molecule in the process of NAFLD development.

Because inflammation has a key role in NAFLD, and knowing that USP13 reduces inflammation in several diseases [16, 17], we further investigated the effects of USP13 on inflammatory pathways in NAFLD. As expected, USP13 inhibited inflammatory pathways, including MAPK and NF-κB signaling pathways, in the livers of NAFLD mice. TAK1 belongs to the MAP3K family and activates the MAPK and NF-κB signaling pathways to significantly modulate several signals [36–38]. It has previously been reported that TAK1 functions as a key activator to mediate autophagy, and regulate inflammation and lipid metabolism in NAFLD [39–42]. In the present study, we observed that USP13 interacted with TAK1 and inhibited its activation. Moreover, inhibiting the phosphorylation of TAK1 reversed the aggravation of hepatic steatosis and inflammation induced by USP13 deficiency. Thus, we proposed that USP13 alleviated NAFLD by targeting TAK1 activation.

Furthermore, how USP13 inhibited TAK1 activation was investigated. It has been shown that K63- and K48-linked polyubiquitination are essential for TAK1 activation [43]. Specifically, the E3 ligase tripartite motif 16 promotes K48-associated phospho-TAK1 ubiquitination and facilitates its degradation, thereby inhibiting the activation of downstream MAPK signaling pathway [44]. In addition, and activation of TAK1 is induced by K63-linked polyubiquitination [23, 45]. USP4 and USP18 bind with TAK1 and de-ubiquitinate TAK1 to inhibit its activation [10, 12]. In the current study, we demonstrated reduced phosphorylation and ubiquitination levels of TAK1 in USP13 overexpression livers of obese mice, but increased phosphorylation and ubiquitination levels in USP13-KO HFD-fed mice. Because TAK1 activation was induced by K63-linked polyubiquitination [23, 45], we assumed that USP13 inhibited TAK1 activation by reducing K63-associated polyubiquitination. Such a hypothesis was verified in USP13-overexpressing L02 cells treated with FFA. Therefore, we deduced that USP13 interacted with TAK1, removed the ubiquitin chain on TAK1, and subsequently reduced TAK1 phosphorylation, followed by activation and downstream events.

Concurrent promotion of liver steatosis, insulin resistance, and inflammation activates pathologic events in metabolic disorders [46]. TAK1 has the ability to regulate JNK pathway jointly with the NF-κB pathway, thus insulin resistance and inflammation provide the primary molecular signaling to TAK1 [45]. The molecular signals associated with IRS modulate insulin function, and the TAK1-JNK1 axis is key in IRS serine/tyrosine phosphorylation, which is associated with insulin sensitivity and resistance [47, 48]. Activation of TAK1 triggers JNK1 to promote IRS1 phosphorylation at serine 307 and inhibits IRS1 tyrosine 608 phosphorylation, which facilitates the interaction between insulin receptors and IRS1, while reducing AKT phosphorylation [21, 49]. As AKT phosphorylation declining, the GSK3β phosphorylation was decreased subsequently that suppresses the synthesis of glycogen and promotes the occurrence of gluconeogenesis by facilitating the expression of PEPCK and G6PC [27]. We showed that the JNK1/2 and p38 MAPK signaling pathway was inhibited and AKT- GSK3β insulin signaling was augmented in USP13 overexpression HFD-fed mice, which illustrated the inhibition ability of insulin resistance and inflammation in USP13 overexpression. Thus, USP13 controlled IRS1 phosphorylation via constraining TAK1–JNK1 axis activation. In addition, the TAK1 downstream IKKβ–P65 axis has been reported to be activated under insulin resistance in addition to liver steatosis [50]; it was shown that activation of the IKKβ–P65 axis was reduced by USP13 overexpression. In order to determinate the necessity of TAK1 for USP13 to alleviate NAFLD, we adopted TAK1 inhibitor to treat USP13-KO mice, and the results indicated that inhibiting the TAK1 activation could neutralize the exacerbation of NAFLD brought from the deficiency of USP13. So, TAK1 activation was pivotal for the mechanism of USP13 to alleviate NAFLD. Therefore, we concluded that USP13 inhibited the JNK/p38 MAPK and NF-κB pathways downstream of TAK1 in addition to its activation and subsequent lowering the inflammatory level and insulin resistance in the liver.

However, there are some limitations in our study. Firstly, we did not determine the specific de-ubiquitination sites on TAK1 by USP13, which warrants further investigation. Secondly, we did not specifically investigate whether the interaction between USP13 and TAK1 changes under HFD versus ND conditions. Lastly, the USP13 overexpression is liver-specific and is not hepatocyte-specific, and future work warrants to be performed.

## Conclusion

In conclusion, our study identifies USP13 as a crucial regulator of insulin resistance and inflammation in NAFLD through its interaction with TAK1. By modulating TAK1 ubiquitination and phosphorylation, USP13 influences key signaling pathways involved in hepatic steatosis and inflammation. These findings suggest that USP13 could be a promising therapeutic target for the treatment of NAFLD. The translational value of our research lies in the potential to develop USP13 modulators that can mitigate liver inflammation and improve insulin sensitivity, thereby addressing the root causes of NAFLD progression. Future research should focus on the development and testing of small molecule inhibitors or activators of USP13 in preclinical models to evaluate their efficacy and safety. Additionally, clinical studies are warranted to assess the relevance of USP13 modulation in human NAFLD patients.

### Supplementary Information

Supplementary Material 1.

## Data Availability

The data of the study are available from the corresponding authors with reasonable request.

## Abbreviations

NAFLD: Nonalcoholic fatty liver disease
USP13: Ubiquitin specific protease 13
FFA: Free fatty acid
HFD: High-fat diet
KO: Knockout
TAK1: Transforming growth factor β-activated kinase 1
NASH: Nonalcoholic steatohepatitis
Ub: Ubiquitin
DUB: De-ubiquitinating enzyme
USPs: Ub-specific proteases
FASN: Fatty acid synthase
HMGCR: 3-Hydroxy-3-methylglutaryl coenzyme A reductase
NF-κB: Nuclear factor kappa B
MAPK: Mitogen-activated protein kinase
cGAS-STING: Cyclic guanosine monophosphate-adenosine monophosphate synthase-stimulator of interferon genes
IL: Interleukin
ND: Normal maintenance diet
GFP: Green fluorescent protein
WT: Wild-type
BSA: Bovine serum albumin
GTT: Glucose tolerance test
ITT: Insulin tolerance test
TC: Total cholesterol
AST: Aspartate aminotransferase
TG: Triglycerides
ALT: Alanine aminotransferase
TNFα: Tumor necrosis factor-alpha
IP: Immunoprecipitation
ACCα: Acetyl coenzyme A carboxylase α
PPAR: Peroxisome proliferator–activated receptor
SREBP-1c: Sterol regulatory element-binding protein-1c
SCD1: Stearoyl-CoA desaturase 1
MCP1: Monocyte chemoattractant protein-1
HOMA-IR: Homeostasis model assessment of insulin resistance
AUC: Area under the curve
FATP: Fatty acid transport protein
FABP: Fatty acid binding protein
CD36: Cluster of differentiation 36
CYP7A1: Cholesterol 7a-hydroxylase 1
ABCG-1: Adenosine triphosphate binding cassette transporter G1
CPT-1α: Carnitine palmitoyltransferase 1
ACOX-1: Acyl-coenzyme A oxidase 1
G6PC: Glucose-6-phosphatase catalytic subunit
PEPCK: Phosphoenolpyruvate carboxykinase
GSK3β: Glycogen synthase kinase-3
AKT: Protein kinase B
IRS1: Insulin receptor substrate 1
*ob/ob*: Leptin-deficient
SPF: Specific pathogen-free
HEK: Human embryonic kidney
BCA: Bicinchoninic acid
H&E: Hematoxylin and eosin
SDS-PAGE: Sodium dodecyl sulfate–polyacrylamide gel electrophoresis
PVDF: Polyvinylidene fluoride
SEM: Standard error of the mean
*P*: Probability
DMSO: Dimethyl sulfoxide
KEGG: Kyoto encyclopedia of genes and genomes
GSEA: Gene set enrichment analysis
