# Supplementary material for: USP13 ameliorates nonalcoholic fatty liver disease through inhibiting the activation of TAK1
Source: J Transl Med. 2024 Jul 20;22:671. doi: 10.1186/s12967-024-05465-4 (PMC11264885; doi:10.1186/s12967-024-05465-4)
Supplement: Supplementary file 1 — Supplementary Material 1. [file 12967_2024_5465_MOESM1_ESM.docx]

**USP13 ameliorates** **nonalcoholic fatty liver disease through inhibiting the activation of TAK1**

Min Tang^1*^, Han Cao ^2,3*^, Yunqin Ma^1*^, Shuangshuang Yao^1^, Xiaohui Wei^1^, Yijiong Tan^4^, Fang liu^1^, Yongde Peng^1,2#^, Nengguang Fan^1#^

^1^Department of Endocrinology and Metabolism, Shanghai General Hospital, Shanghai Jiao Tong University School of Medicine, Shanghai, China;

^2^Department of Endocrinology and Metabolism, Shanghai General Hospital of Nanjing Medical University, Shanghai, China;

^3^Department of Endocrinology, Songjiang District Central Hospital, Shanghai, China;

^4^Department of Clinical Pharmacy, Shanghai General Hospital, Shanghai Jiao Tong University School of Medicine, Shanghai, China;

*These authors contributed equally to this work.

**^#^**Correspondence:

Corresponding Author: Nengguang Fan, [fngwlp@163.com](mailto:fngwlp@163.com); Yongde Peng, [yongdepeng0908@126.com](mailto:pengyongde0908@126.com);

**Supplementary Table 1. Primers used for RT-qPCR.**

| Gene | Forward (5’-3’) | Reverse (5’-3’) |
| --- | --- | --- |
| Mouse |  |  |
| GAPDH | CCATGTTCGTCATGGGTGTGAACCA | GCCAGTAGAGGCAGGGATGATGTTC |
| ABCG-1 | GTACCATGACATCGCTGGTG | AGCCGTAGATGGACAGGATG |
| CYP7A1 | ACACCATTCCTGCAACCTTC | GCTGTCCGGATATTCAAGGA |
| HMGCR | TGGAGATCATGTGCTGCTTC | GCGACTATGAGCGTGAACAA |
| CD36 | AAGATGACGTGGCAAAGAACAG | CCTTGGCTAGATAACGAACTCTG |
| FABP1 | TACCAATTGCAGAGCCAGGA | ACTCATTGCGGACCACTTTG |
| FATP1 | CTACCACTCTGCAGGGAACA | CAGGTAGCGGCAGATTTCAC |
| FATP5 | TTTCTGGGGTTGGCCAAGTT | TGGCCAAGGTAGAAGCAGTG |
| FASN | GGAGGTGGTGATAGCCGGTAT | TGGGTAATCCATAGAGCCCAG |
| SREBP-1c | CAAGGCCATCGACTACATCCG | CACCACTTCGGGTTTCATGC |
| ACCα | GATGAACCATCTCCGTTGGC | GACCCAATTATGAATCGGGAGTG |
| PPARγ | ATTCTGGCCCACCAACTTCGG | TGGAAGCCTGATGCTTTATCCCCA |
| PPARα | TTTCGGCGAACTATTCGGCTG | GGCATTTGTTCCGGTTCTTCTT |
| CPT-1α | AGATCAATCGGACCCTAGACAC | CAGCGAGTAGCGCATAGTCA |
| ACOX-1 | GCACCATTGCCATTCGATACA | CCACTGCTGTGAGAATAGCCGT |
| PEPCK | CTGCATAACGGTCTGGACTTC | CAGCAACTGCCCGTACTCC |
| G6PC | GACCTCCTGTGGACTTTGGA | AGTTCTCCCTTGCAGCTCTT |
| IL-1β | GCAACTGTTCCTGAACTCAACT | ATCTTTTGGGGTCCGTCAACT |
| IL-6 | CCACTTCACAAGTCGGAGGCTTA | GCAAGTGCATCATCGTTGTTCATAC |
| TNFα | AGCCCCCAGTCTGTATCCTT | CTCCCTTTGCAGAACTCAGG |
| MCP1 | CTTCTGGGCCTGCTGTTCA | CCAGCCTACTCATTGGGATCA |
| Human |  |  |
| GAPDH | CCATGTTCGTCATGGGTGTGAACCA | GCCAGTAGAGGCAGGGATGATGTTC |
| SREBP-1c | CGGAGCCATGGATTGCACTTTC | GATGCTCAGTGGCACTGACTCTCC |
| SCD1 | TTGATTCCTGGCTCTACCC | TCACTGCCTCTGAATACACA |
| ACCα | TTGATTCCTGGCTCTACCC | TCACTGCCTCTGAATACACA |
| PPARγ | TTGCAGTGGGGATGTCTCAT | TTTCCTGTCAAGATCGCCCT |
| IL-1β | CCACGGCCACATTTGGTT | AGGGAAGCGGTTGCTCATC |
| IL-6 | TCAATGAGGAGACTTGCCTGGT | TACTCATCTGCACAGCTCTGGCT |
| TNFα | AGCTGGTGGTGCCATCAGAGG | TGGTAGGAGACGGCGATGCG |
| MCP1 | CAGCCAGATGCAATCAATGCC | TGGAATCCTGAACCCACTTCT |

**Supplementary Table 2. Antibodies used for the study.**

| Antibody | Source | Cat number | Dilution rate |
| --- | --- | --- | --- |
| USP13 | Abcam | ab109264 | 1：1000 |
| GAPDH | Cell Signaling Technology | 5174S | 1：2000 |
| HNF4 | Abcam | ab41898 | 1：2000 |
| TAK1 | Cell Signaling Technology | 4505 | 1：1000 |
| TAK1 | Proteintech | 12330-2-AP | 1：1000 |
| phosphorylated-TAK1 | Invitrogen | PA5-99340 | 1：250 |
| phosphorylated-TAK1 | Cell Signaling Technology | 4531 | 1：250 |
| P65 | Cell Signaling Technology | 8242T | 1：1000 |
| phosphorylated-P65 | Cell Signaling Technology | 3033T | 1：1000 |
| IKKβ | Cell Signaling Technology | 8943T | 1：1000 |
| phosphorylated-IKKα/β | Cell Signaling Technology | 2697T | 1：1000 |
| p38 | Cell Signaling Technology | 8690T | 1：1000 |
| phosphorylated-p38 | Cell Signaling Technology | 4511T | 1：1000 |
| JNK | Cell Signaling Technology | 9252T | 1：1000 |
| phosphorylated-JNK | Cell Signaling Technology | 4668T | 1：1000 |
| AKT | Cell Signaling Technology | 4691 | 1：1000 |
| phosphorylated-AKT | Cell Signaling Technology | 4060 | 1：1000 |
| IRS1 | Cell Signaling Technology | 3407 | 1：1000 |
| phosphorylated-IRS1 | Cell Signaling Technology | 2381 | 1：1000 |
| GSK3β | Cell Signaling Technology | 12456T | 1：1000 |
| phosphorylated-GSK3β | Cell Signaling Technology | 5558T | 1：1000 |
| Ub | Cell Signaling Technology | 3936S | 1：1000 |
| Ub (linkage-specific K63) | Millipore | 05-1308 | 1：1000 |
| HA | Proteintech | 51064-2-AP | 1：1000 |
| Flag | Proteintech | 20543-1-AP | 1：1000 |
| MYC | Proteintech | 60003-2-lg | 1：1000 |
| HRP-conjugated anti-rabbit secondary antiboy | Cell Signaling Technology | 7074 | 1：5000 |
| HRP-conjugated anti-mouse secondary antiboy | Abcam | ab6789 | 1：5000 |

**
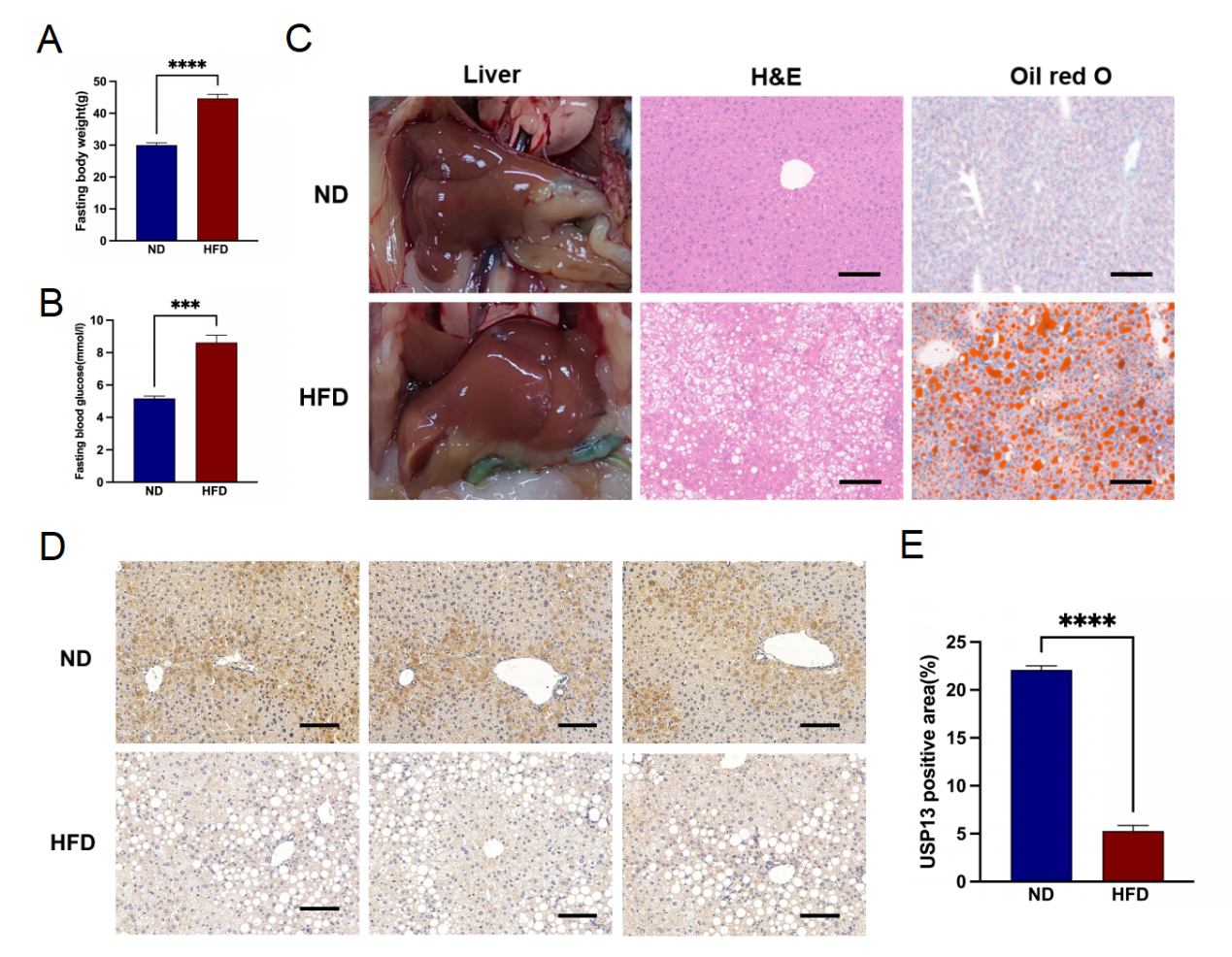
**

**Supplementary Figure 1.** USP13 expression levels in livers of ND-fed mice and HFD-fed mice. (A) The fasting body weight of ND-fed mice and HFD-fed mice. (B)The fasting blood glucose of ND-fed mice and HFD-fed mice. (C) The liver appearance, H&E and Oil Red O staining of liver sections in ND-fed mice and HFD-fed mice. (D) Immunohistochemistry suggests decreased expression level of USP13 in the liver of HFD-fed mice.


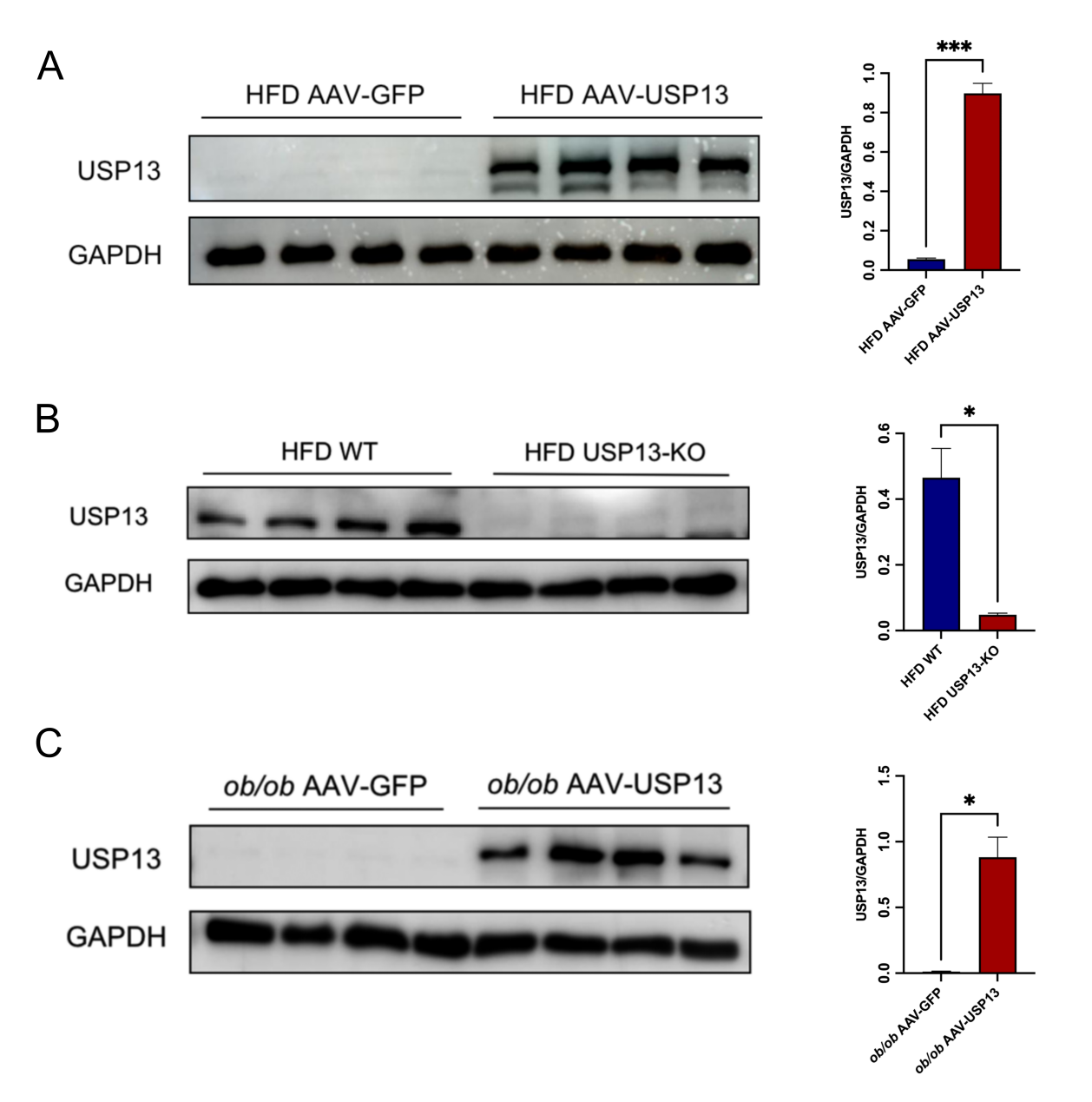


**Supplementary Figure 2.** Western blot analysis of USP13 expression levels in livers of mice. (A)Western blot analysis of USP13 expression levels in livers of HFD AAV-GFP and HFD AAV-USP13 mice. (B) Western blot analysis of USP13 expression levels in livers of HFD WT and HFD USP13-KO mice. (C) Western blot analysis of USP13 expression levels in livers of *ob/ob* AAV-GFP and *ob/ob* AAV-USP13 mice.

**
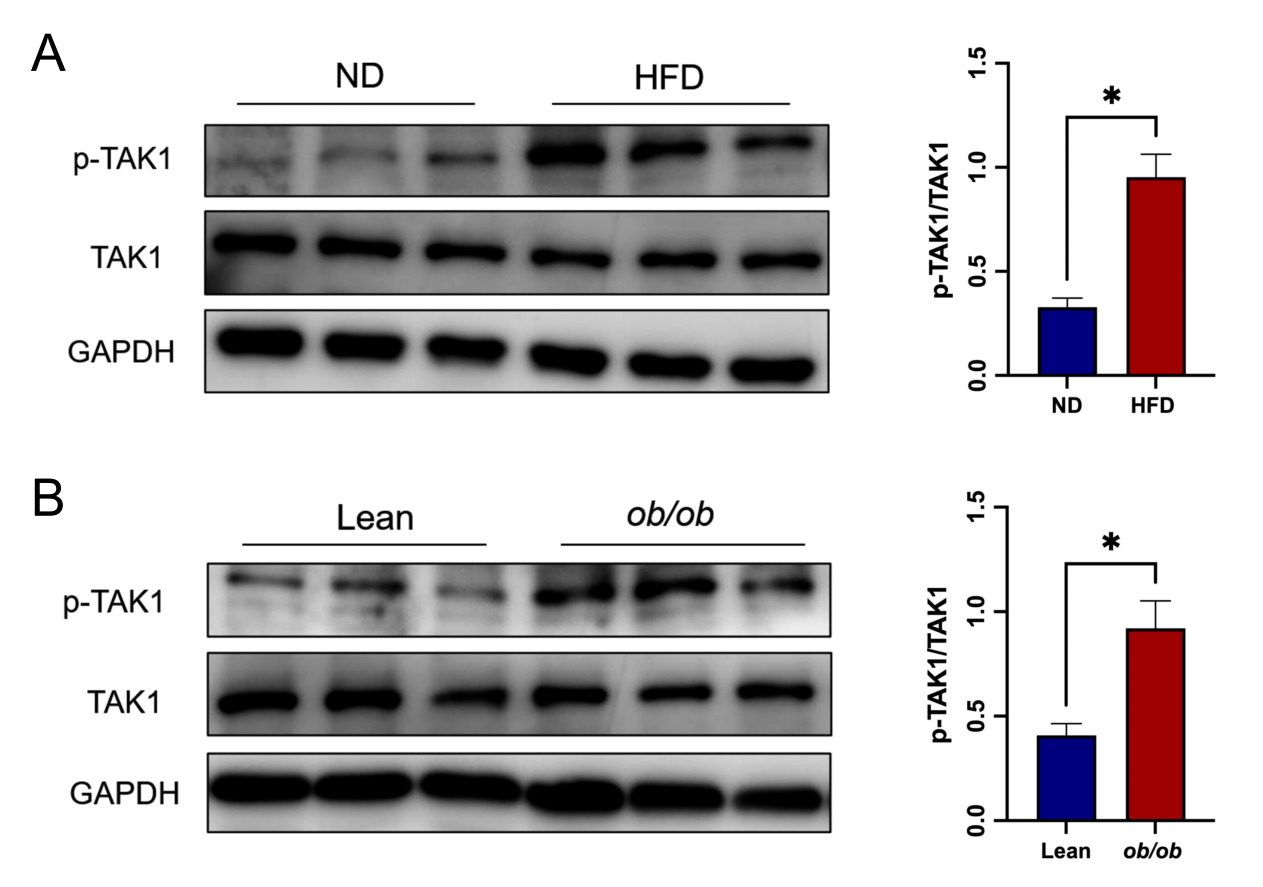
**

**Supplementary Figure 3.** Western blot analysis of p-TAK1 expression levels in livers of mice. (A)Western blot analysis of p-TAK1 expression levels in livers of ND and HFD mice. (B) Western blot analysis of p-TAK1 expression levels in livers of Lean and *ob/ob* mice.
